# Nitrate-responsive *OBP4-XTH9* regulatory module controls lateral root development in *Arabidopsis thaliana*

**DOI:** 10.1371/journal.pgen.1008465

**Published:** 2019-10-18

**Authors:** Peipei Xu, Weiming Cai

**Affiliations:** Laboratory of Photosynthesis and Environment, CAS Center for Excellence in Molecular Plant Sciences, Shanghai Institute of Plant Physiology and Ecology, Chinese Academy of Sciences, Shanghai, China; Peking University, CHINA

## Abstract

Plant root system architecture in response to nitrate availability represents a notable example to study developmental plasticity, but the underlying mechanism remains largely unknown. Xyloglucan endotransglucosylases (XTHs) play a critical role in cell wall biosynthesis. Here we assessed the gene expression of *XTH1-11* belonging to group I of XTHs in lateral root (LR) primordia and found that *XTH9* was highly expressed. Correspondingly, an *xth9* mutant displayed less LR, while overexpressing *XTH9* presented more LR, suggesting the potential function of *XTH9* in controlling LR development. *XTH9* gene mutation obviously alters the properties of the cell wall. Furthermore, nitrogen signals stimulated the expression of *XTH9* to promote LRs. Genetic analysis revealed that the function of *XTH9* was dependent on auxin-mediated *ARF7/19* and downstream *AFB3* in response to nitrogen signals. In addition, we identified another transcription factor, *OBP4*, that was also induced by nitrogen treatment, but the induction was much slower than that of *XTH9*. In contrast to *XTH9*, overexpressing *OBP4* caused fewer LRs while *OBP4* knockdown with *OBP4-RNAi* or an artificial miRNA silenced *amiOBP4* line produced more LR. We further found OBP4 bound to the promoter of *XTH9* to suppress *XTH9* expression. In agreement with this, both *OBP4-RNAi* and crossed *OBP4-RNAi* & *35S*::*XTH9* lines led to more LR, but *OBP4-RNAi* & *xth9* produced less LR, similar to *xth9*. Based on these findings we propose a novel mechanism by which *OBP4* antagonistically controls *XTH9* expression and the *OBP4*-*XTH9* module elaborately sustains LR development in response to nitrate treatment.

## Author summary

Nitrate is not only a nutrient, but also a signal that controls downstream signaling genes at the whole-plant level. In plants, changes in root system architecture in response to nitrate availability represent a notable example of developmental plasticity in response to environmental stimuli. However, the molecular mechanisms underlying nitrate-associated modulation are largely unknown. Here, we identified a nitrogen-responsive signaling module that comprises both xyloglucan endotransglucosylase 9 (XTH9) and the Dof transcription factor OBP4 and controls lateral root (LR) development. We used root gravitropic bending assays to observe the gene expression of group 1 xyloglucan endotransglucosylases (XTHs) involved in LR primordia. The results showed that *XTH9* expression patterns were changed and that *xth9* knockout mutants displayed altered LR growth. *XTH9* was expressed in the LRs and in response to nitrate treatment, and the *xth9* mutants were defective in nitrate-promoted LR growth. Moreover, *XTH9* overexpression increased LR length and increased tolerance to low-nitrate stress. We found that *OBP4* could negatively regulate *XTH9* and inhibited root growth. *OBP4* and *XTH9* worked downstream of ARF7/9. We conclude that *OBP4* and *XTH9* constitute a regulatory module which contributes to LR growth in response to different environmental nitrate concentration signals.

## Introduction

In the biosphere, nitrate is the major form of nitrogen, and nitrate availability is important for plant development. Nitrate is not only a nutrient, but also a signal that controls downstream signaling genes at the whole-plant level [1, 2]. Previous genomic studies have shown that the nitrate response is comprehensive, but the molecular mechanisms of nitrate signal transduction and the downstream gene expression changes that lead to developmental responses, such as changes in root system architecture (RSA), are still unclear. In the last few years, several nitrate regulatory genes functioning in the primary nitrate response have been characterized. One key regulator is nitrate transporter 1.1 (*NRT1*.*1*), which functions not only as a dual-affinity nitrate transporter, but also as a nitrate sensor [3–5]. The CBL-interacting protein kinases, CIPK8 and CIPK23, are involved in the primary nitrate response [6–8]. Similarly, the MADS family NO3^−^-inducible transcription factor (TF) *ANR1* and Lateral Organ Boundaries Domain (LBD) regulate lateral root (LR) growth in response to nitrate treatment [8, 9]. NIN-like protein 7 (NLP7) orchestrates the early response to nitrate in plants [10], and via a systems biology approach, TGACG SEQUENCE -SPECIFIC BINDING PROTEIN 1(*TGA1*), AUXIN SIGNALING F-BOX 3 (*AFB3*), ARABIDOPSIS NAC DOMAIN-CONTAINING PROTEIN 79 (*NAC4*), and *OBP4* genes have been identified as nitrate regulators involved in nitrate signaling [2,11,12].

In *Arabidopsis*, RSA plays a role in nutrient and water absorption, which is important for plant growth and development. Thus, plants require modulation of RSA plasticity to respond to various dynamic environmental conditions. RSA is a plastic characteristic in many plant species, but the plasticity of the RSA phenotype is controlled via inherent genetic mechanisms. Lateral root primordia (LRP) are derived from many primary root pericycle cells. New LRs originate from LRP, followed by several rounds of cell division [13]. LRP play a critical role in the initial stages of LR initiation [14]. LRs serve as a fascinating model for determining how adjacent cells synchronize growth and development [15, 16]. The cell cycle inhibitor Kip-associated gene 2 exhibits important gene functions in the cycling of pericycle cells and blocks the G1-to-S phase transition [17]. In a more distal region, protoxylem pericycle cells proceed to the G2 phase and become competent for LR initiation [18, 19]. Furthermore, several important studies linking cell wall modifications and LR development have been published [20, 21], and several studies have also reported a role for xyloglucan endotransglucosylases (XTHs) during LR development [20, 22].

Auxin is an important hormone in LR development [23]. To emerge, the LRP must break through the overlying tissues (including endodermal, cortical, and epidermal cells). Development subsequently progresses via auxin signaling reprogramming of overlying tissues to undergo cell separation, promoting the emergence of the LRs [20, 24–27]. Auxin signaling during the formation of LRP is associated with protein degradation. First, auxin binds to AFB/TIR1 receptors and mediates their association with and the degradation of indoleacetic acid (IAA) proteins. Furthermore, auxin-induced Aux/IAA degradation enables auxin-response factors (ARFs) to transactivate the transcription of downstream genes [28]. Both Aux/IAA proteins and ARF transcriptional regulators are critical factors for sensing the concentration of auxin and subsequently translating that signal into the expression of downstream genes, further influencing the output of patterning in plant growth and development [29, 30].

XTHs play pivotal roles in the reconnection and splitting of xyloglucan crosslinks by catalyzing the molecular grafting of the xyloglucans to form a framework [31–33]. XTH also plays important roles in plant cell wall construction and disassembly [33]. XTHs compose a large gene family in multiple plant species. A total of 33 XTH genes have been identified in *Arabidopsis*, and one-third of them occur as clusters, potentially reflecting genomic duplication events [34]. Every member of the XTH gene family is likely controlled by specific cues that respond to the cell wall dynamics of certain tissues and/or cell types [35]. For example, *AtXTH27* is crucial for tertiary vein development in rosette leaves and is also involved in cell wall modification of tracheary elements [36], and the function of *AtXTH31* is important during cell elongation under Al stress because it modulates XTH activity [37]. Comprehensive analyses of *XTH* gene expression have revealed that many of these enzymes are expressed in the roots [38]. Despite these studies, little is known about the role of *XTH* genes in nitrate signaling with respect to the regulation of root system development.

In the present study, to determine whether *XTH* genes are responsible for LR development, we used a reverse genetics method to analyze the root development phenotypes of class 1 *XTH* genes. We subsequently identified an *XTH9* mutation that causes a defect in LR development. Analysis of proXTH9 fused to GUS revealed that *XTH9* is expressed in the LRs in response to nitrate treatment, and auxin regulates *XTH9* expression in the roots via *ARF7*/*19*. Furthermore, we observed that the nitrate regulation of *XTH9* occurs downstream of the AFB3-IAA14 signaling pathway. We also identified a Dof TF, OBP4, that negatively regulates *XTH9*. *OBP4* was reported to be regulated by nitrate directly as a signal, and systems approaches showed that it was acting downstream of AFB3 [2, 12]. The *OBP4* gene fine-tunes LR development in response to environmental nitrate availability. *OBP4* and *XTH9* constitute a regulatory module which controls LR growth in response to variation in nitrate concentrations. Thus, we deduce that the *OBP4*-*XTH9* regulatory module has a specific function in LR development in response to nitrate signaling in *Arabidopsis*.

## Results

### Expression of group I *XTH* genes in LRP

The XTH/hydrolyase enzyme plays a role in the endo-transglucosylation and/or hydrolysis of xyloglucan molecules. In the *Arabidopsis* genome, 33 genes encoding XTH proteins have been isolated. Individual members of this gene family exhibit specific expression patterns, both temporally and spatially. Most of the *XTH* genes are functionally redundant, but some members have roles in specific aspects of plant growth and development. Among these members, 11 (*XTH1-11*) cluster into group I [38–40]. Because the expression patterns of many group I *XTH* genes occur in the roots, we initially investigated whether the expression of the cell wall modification-related *XTH* gene changed during LR development.

**Fig 1:**
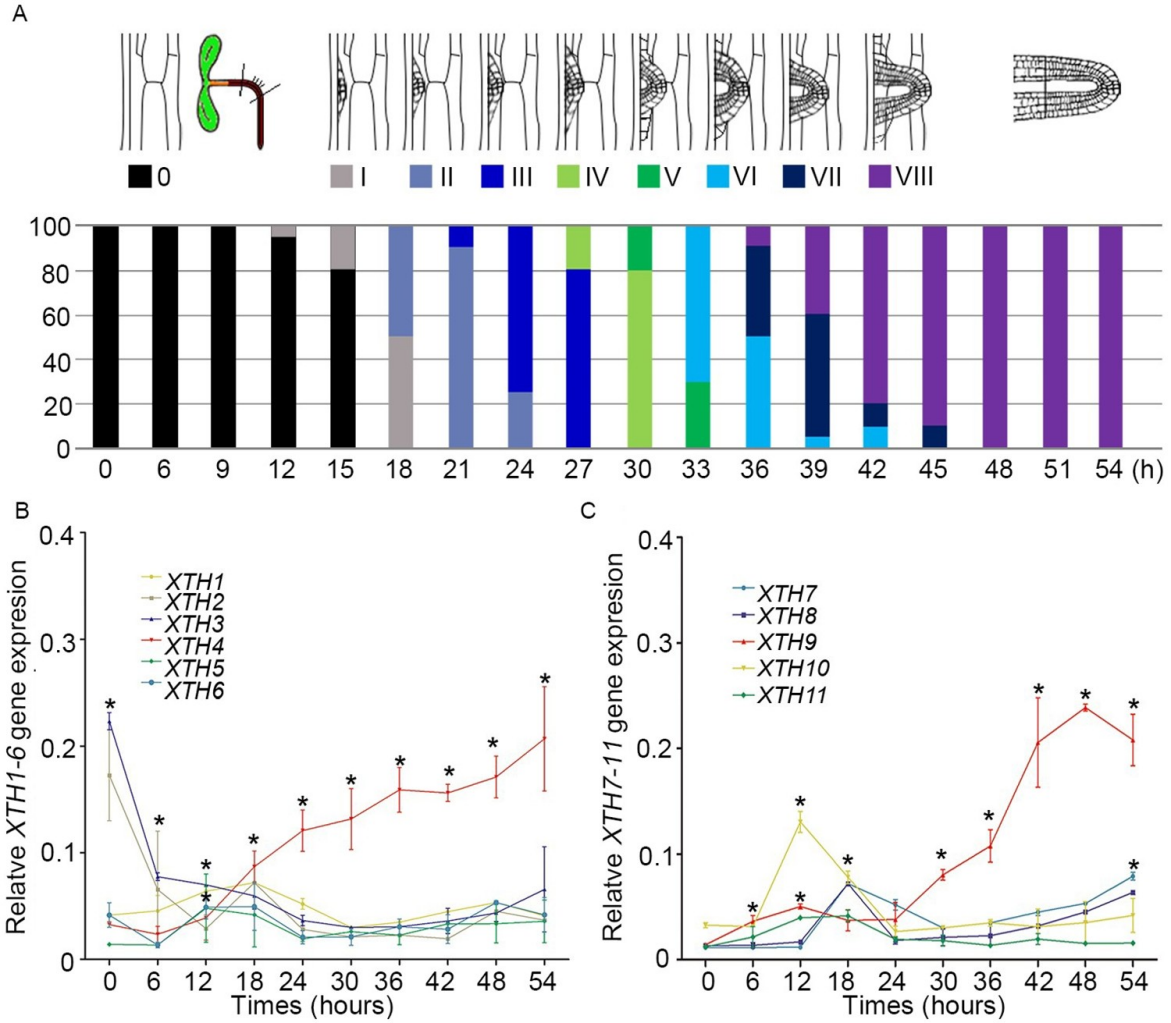
Transcriptomic analysis of class 1 *XTH* genes during lateral root (LR) initiation. (A) After gravitropic stimulation to induce the synchronized initiation of lateral root primordia (LRP) at the surface of bending roots, LRP stages from I to VIII, in accordance with previous reports, were assessed every 6 hours for 0 to 54 hours after gravity stimulation and are represented here as a percentage of the total number of observed LRP at each time point. At least 70–80 LRP were observed at each time point. (B, C) All class 1 *XTH* gene expression patterns at each time point during LR initiation were measured every 6 hours from 6 to 54 hours pgi. Bending roots of a population of 5-day-old seedlings were microdissected at 10 time points, which were then used for RNA extraction (approximately 220 per time point for three independent replicates). Expression patterns of all class 1 *XTH* genes are shown. The error bars denote SDs (n = 4). Student’s *t*-test was applied. The asterisks (*) show statistically significant differences (*p*<0.05).

Both mechanical and/or gravitropic stimuli can facilitate the initiation of LRs [41–44]. LR initiation occurs in a highly synchronized manner on the outer surface of a bending root and can be measured with 90° gravitropic stimulus assays [27, 45]. We can classify this developmental progress into many different stages. In stage I, LRP was observed at 18 hours post-gravitropic induction (pgi), and subsequently, at approximately 6-hour intervals, each subsequent primordium stage was measured until the LR emerged at 42 hours pgi in stage VIII (Fig 1A). Because we measured the microdissected root bends every 6 hours pgi, we profiled *XTH* gene expression at all stages of LR development (Fig 1A). In this study, five-day-old Col-0 plants grown under normal conditions were used for gravity stimulus assays, and then the transcriptional profiles of the 11 XTHs were measured. We found increased expression levels of *XTH4* and *XTH9* during LR development. *XTH10* gene expression was strongly increased during the first 12 hours after treatment and then sharply decreased (Fig 1B and 1C). The transcriptional level of other genes in group I did not present a dramatic change, suggesting the potential function of *XTH4*, *XTH9*, or *XTH10* during LR initiation. To verify the accuracy of our experimental system, we further assessed the transcript level of the well-known LR regulator *LBD29*. Consistent with the previous study (S1 Fig) [46, 47], the transcriptional level of *LBD29* was substantially upregulated at the indicated time points.

### *XTH9* mutation decreased LR density

**Fig 2:**
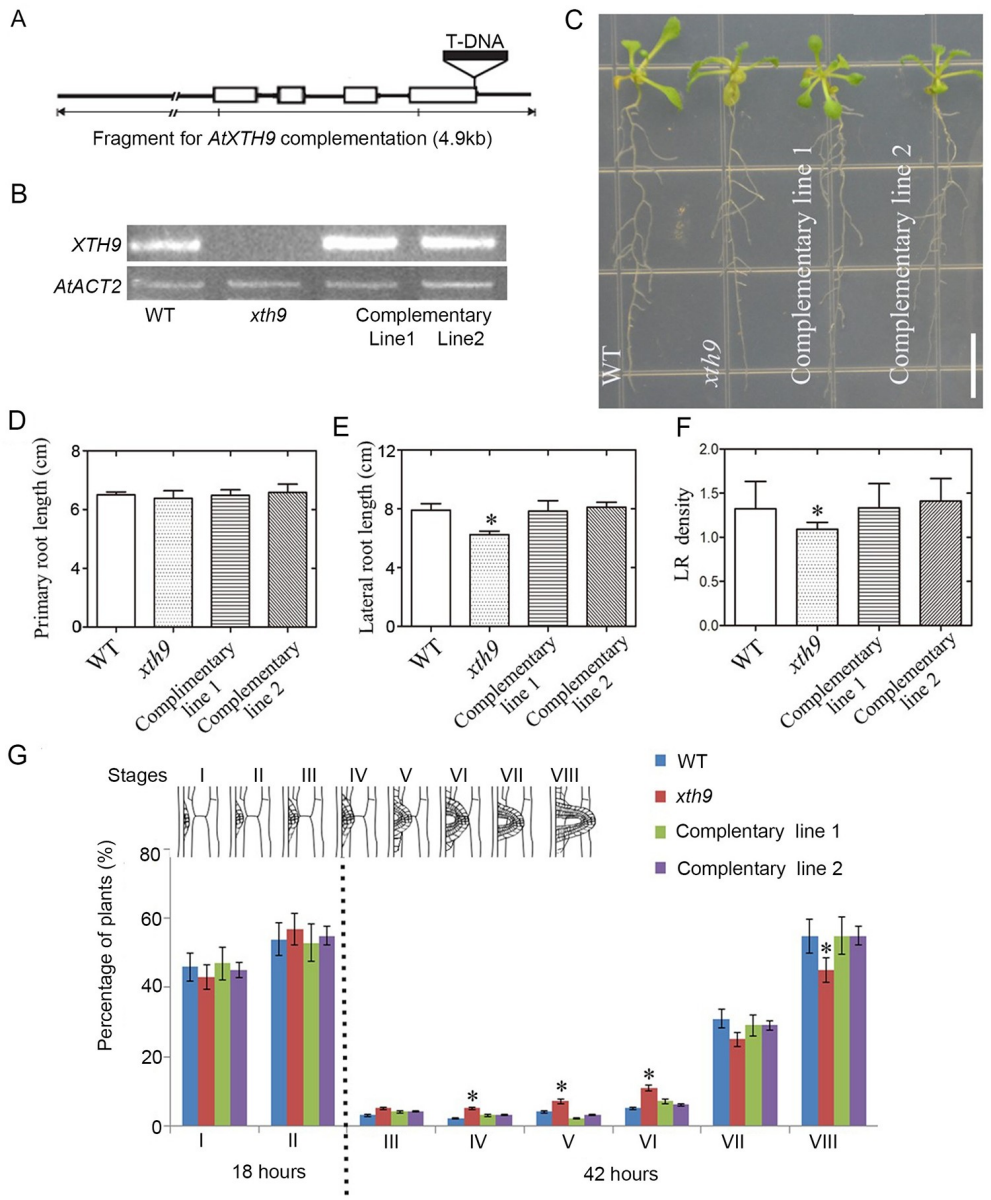
*XTH9* gene mutation affects lateral root (LR) development. (A) The genomic organization of the *XTH9* gene. The white boxes show the positions and sizes of the *XTH9* gene exons. The black box indicates the structure of the T-DNA, the site of which is indicated by the triangle. The genomic sequences used to complement the *xth9* mutation are underlined with a thick line. (B) *XTH9* transcript levels in wild-type (WT) (Col-0) plants, the *xth9* mutant, and *xth9* complementary lines. The mRNA abundance of the *XTH9* gene in the roots was measured using RT-PCR in various genotype backgrounds of *Arabidopsis*. (C) Root phenotypes of WT plants, the *xth9* mutant, and complementary lines. The plants were grown vertically on media for 10 days. The white bar indicates 1 cm. (D) Primary root length and (E) total LR length. (F) LR density analysis of WT plants, the *xth9* mutant, and *xth9* complementary lines. The error bars denote SDs (n = 16–21). (G) Phenotypic analysis of lateral root emergence (LRE) was achieved by synchronizing LR formation with a gravity stimulus for 18 and 42 hours. Compared with the WT plants, the *xth9* mutants showed delayed LRE. Mutant plants transformed with a 4.9-kb-long full-length *XTH9* genomic fragment exhibited a WT LRE phenotype for LR induction. The data are shown as percentages, and the error bars represent SDs (n = 15–17). At least 80–100 total LR primordia were observed for each plant, and the asterisks (*) indicate statistically significant differences (*p*<0.05).

The expression patterns of *XTH* genes were altered during root bending under gravity stimuli; therefore, we sought to obtain further insight into the roles of the group I XTH family member, XTH1-11. We isolated and analyzed the phenotypes of homogeneous T-DNA insertion lines to observe the LR phenotypes of all 11 group I *XTH* genes (S2A Fig & S3 Table). Among these lines, the SALK_101024 line (named *xth9*) had a T-DNA insertion in the 3′ UTR of *XTH9* (Fig 2A). After assessing the mRNA level of *XTH9* in this mutant background, we determined that these plants represented a knockout line (Fig 2B). We also assessed other T-DNA insertion lines of *XTH9* (SALK_063401, SALK_002571, and SALK_023274). These mutations were unable to downregulate *XTH9* expression, which was likely due to all the T-DNA inserts detected in the promoter region of *XTH9*. After careful observation, we discovered that the *xth9* mutant exhibited defective LR growth. However, no obvious root phenotype was observed in *XTH4*, *XTH10* (S2B and S2C Fig) or the other group I *XTH* gene mutants. Therefore, we focused on *XTH9*. Under the experimental growth conditions, this gene mutation reduced LR development, but did not affect main root development (Fig 2C–2F). Furthermore, we used the full-length 4.9-kb genomic DNA of *XTH9* to complement the mutant phenotype, and the results showed that the transgenic lines exhibited LR development that was similar to that of the wild-type (WT; Col-0) (Fig 2C). Next, the WT and mutant roots were exposed to a gravitropic stimulus, and the LRP were counted and staged at 18 and 42 hours pgi. The WT plants accumulated stage I and II LRP at 18 hours pgi and stage VII and VIII LRP at 42 hours pgi. However, compared with the WT, the *xth9* mutant exhibited a higher percentage of stage III-VI LRP and a lower accumulation of stage VII-VIII LRP at 42 hours pgi (Fig 2G). The lateral root emergence (LRE) of the complemented plants was consistent with that of the WT plants (Fig 2G). Altogether, these results suggested that *XTH9* positively regulates LR development; thus, we further examined the molecular mechanism underlying this phenotype.

Since XTHs are involved in hemicellulose regulation, we tested the hemicellulose content of *xth9* mutant. The total residual sugar in hemicellulose was reduced in *xth9* mutant (S3A Fig). XTH can cleave or rejoin xyloglucan (XyG) chains, resulting in modified content or structure of the XyG, and XyG is shown to regulate the ability of cell wall extension in response to environmental changes. We analyzed the xyloglucan content in the root cell wall by enzyme digestion and MALDI-TOF analysis. The overall XyG repeat was obviously reduced in *xth9* mutant, especially XXXG, XXFG, XLFG, and XXLG (S3B Fig). These results suggest that mutations in the *XTH9* alter the properties of cell wall. The Group II XTH gene family has XET activities, we found that the XET activity of *xth9* mutant was reduced under our growth conditions by using endogenous xyloglucan as the donor substrate (S3D Fig). These data indicate that the XET action in the roots requires *XTH9*.

### *XTH9* expression in the LRs and in response to nitrate treatment

**Fig 3:**
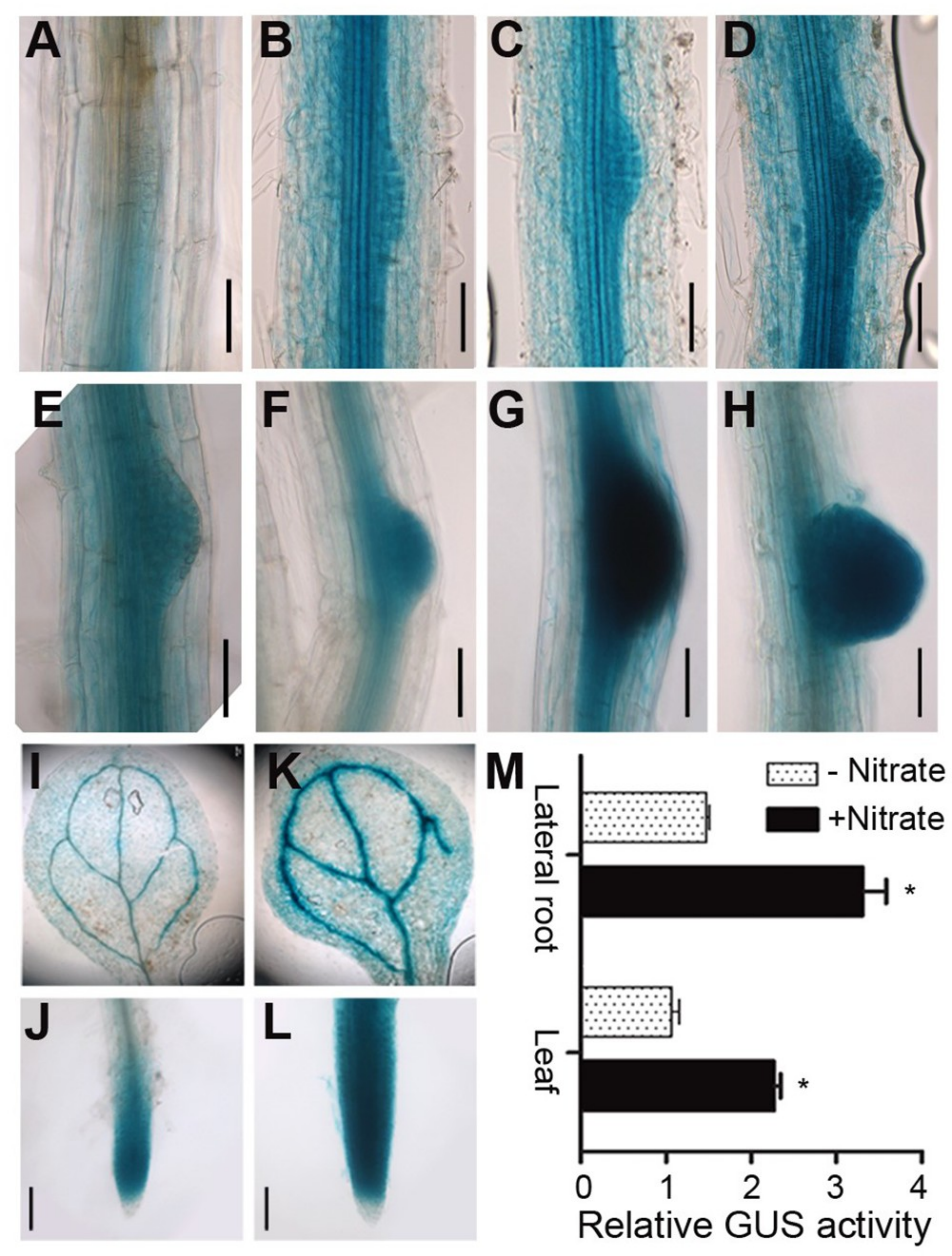
Observation of proXTH9::GUS transgenic lines during lateral root (LR) development. (A–H) GUS staining in proXTH9::GUS *Arabidopsis* roots at various developmental stages. The seedlings were incubated on media supplemented without (I, J) or with (K, L) 5 mM nitrate for 6 hours. Images of the leaves (I, K) and LRs (J, L) were obtained, and representative images are shown. (M) Relative GUS activity before and after nitrate treatment is shown. Leaf GUS activity in the absence of nitrate was used as a control. The asterisks (*) show statistically significant differences (*p*<0.05) and the error bars represent SDs (n = 4).

Given the upregulation of *XTH9* in the 90° gravitropic stimulus assay and the decreased LR growth in the *xth9* mutant, RT-qPCR was used to examine the *XTH9* mRNA levels in various plant organs, including shoots, flowers, roots, buds, and rosette leaves, as well as in seedlings. *XTH9* was expressed in all the organs examined and relatively high expression levels were detected in seedlings, flowers, and roots (S3C Fig). Furthermore, we generated a *proXTH9*::*GUS* transgenic line driven by its 2.3-kb native promoter. We obtained 11 independent transgenic lines, and we selected three of them to observe GUS signals in the LRP. GUS signals were observed during LR initiation and elongation in all these lines. *XTH9* expression was observed across the entire LR (Fig 3A–3H). This expression pattern suggests an important role for this gene in root development. To further identify *XTH9* nitrate responses and understand their involvement in LR growth, we performed an *XTH9* expression survey in *Arabidopsis* roots. First, we checked the expression of *XTH9* in response to various abiotic stresses (mannitol, heat, and salt), nutrients (nitrogen and phosphorus), and hormones (ABA and GA). The results showed that *XTH9* expression increased in response to nitrate treatments (S4 Fig). Therefore, we focused on *XTH9*-mediated LR formation and nitrate signaling. Nitrate significantly affects LR development [48], and we observed that nitrate treatment promoted *XTH9* expression (Fig 3I–3M). Additional transcript profiling and reporter studies demonstrated that *XTH9* is expressed in response to nitrate treatment. We concluded that the *XTH9* gene regulates LR development in response to nitrate signals.

### The *xth9* mutant was defective in nitrate-promoted LR growth

**Fig 4:**
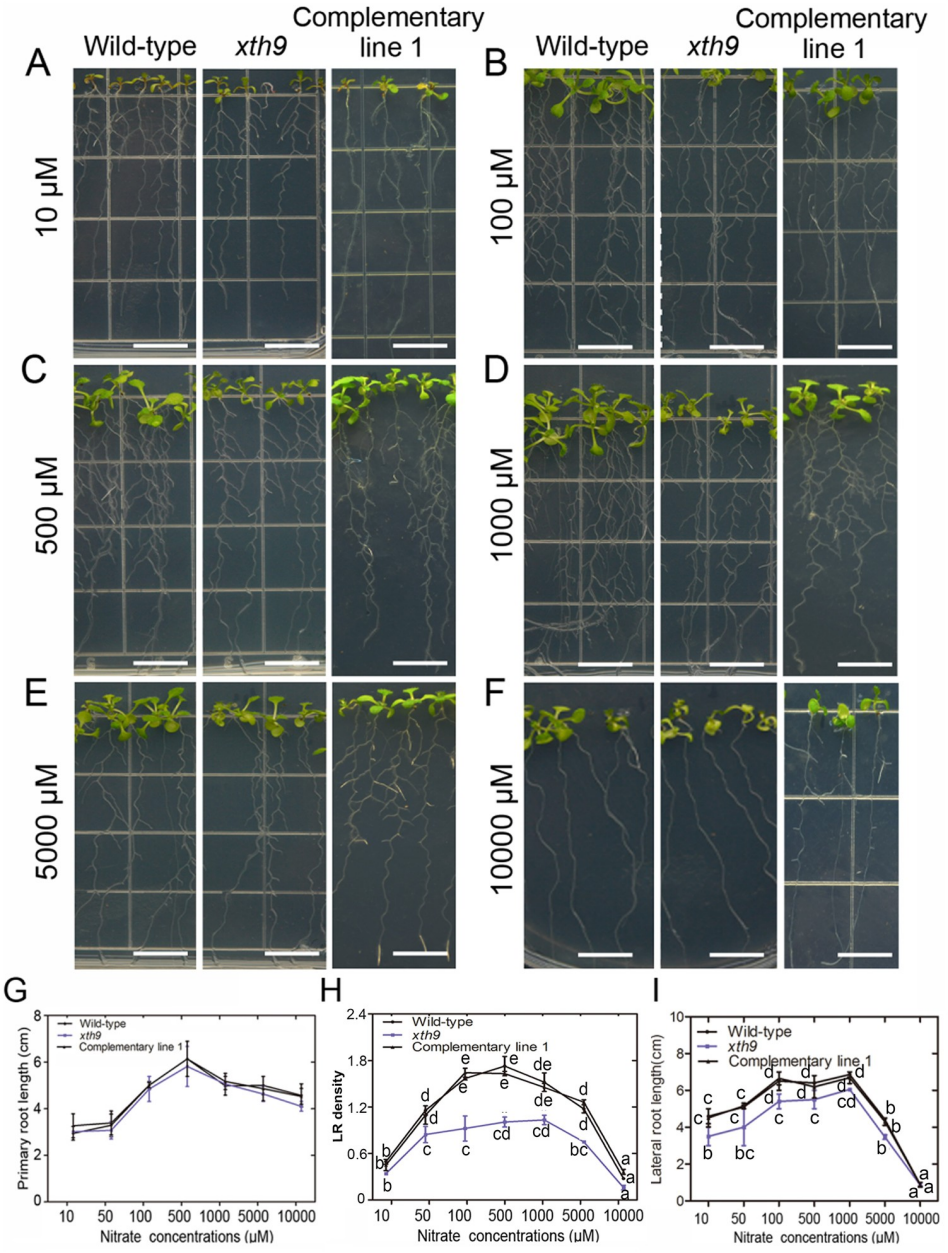
*XTH9* gene mutation altered lateral root (LR) development in response to nitrate treatments. (A–F) Effects of various concentrations of nitrate (10, 50, 100, 500, 1000, 5000, and 10000 μM) on root growth. WT, *xth9* mutant, and the complementary line 1 were grown vertically in media supplemented with various nitrate concentrations for 8 days. (G) Primary root length; (H) LR density; (I) Lateral root length were quantified. LR density was calculated by dividing the LR number per 1 cm of primary root. The error bars represent SDs (n = 22–31). Bars with different capital letters indicate significant differences among different concentrations of nitrate treatment in the given genotype by ANOVA analysis. The white bars indicate 1.2 cm.

Furthermore, datasets involving various concentrations of N-supplied roots were analyzed. We conducted RT-qPCR analysis of *XTH9* in the roots of 12-day-old Col-0 *Arabidopsis* seedlings grown under various concentrations of nitrate (Fig 4).

The effects of nitrate on LR growth depended on the concentration. Moderate concentrations promoted LR growth, but low or high levels of nitrate inhibited LR growth [49]. We then investigated the root development of WT and *xth9* mutant plants on media supplemented with various concentrations (10, 50, 100, 500, 1000, 5000, and 10000 μM) of nitrate. The results showed that, under N-deficient conditions (10 μM nitrate), both the WT and *xth9* plants showed N-deficient phenotypes with strongly inhibited LR initiation. Additionally, the *xth9* plants had fewer LRs than the WT plants (Fig 4A, 4H and 4I). When the nitrate concentration increased to 100–1000 μM, these moderate concentrations of nitrate substantially promoted LR growth in WT plants, but the *xth9* plants were insensitive to these nitrate concentrations. Moreover, the LR density and total root length were also lower in the *xth9* plants than in the WT control plants (Fig 4B–4D, 4H and 4I). High nitrate concentrations (5000–10000 μM) inhibited LR initiation in both the WT and *xth9* mutant plants (Fig 4E–4I). In response to increasing nitrate concentrations, the LR density and total LR length curve for the *xth9* mutant plants was lower than that for WT plants (Fig 4H and 4I). However, in the complementary transgenic lines, the phenotype recovered to that of the WT (S5 Fig). Therefore, in the *xth9* mutant, nitrate-promoted LR growth was defective, but the main root elongation was not substantially affected (Fig 4G).

### *XTH9* gene overexpression increased LRs and enhanced tolerance to low-nitrate stress

**Fig 5:**
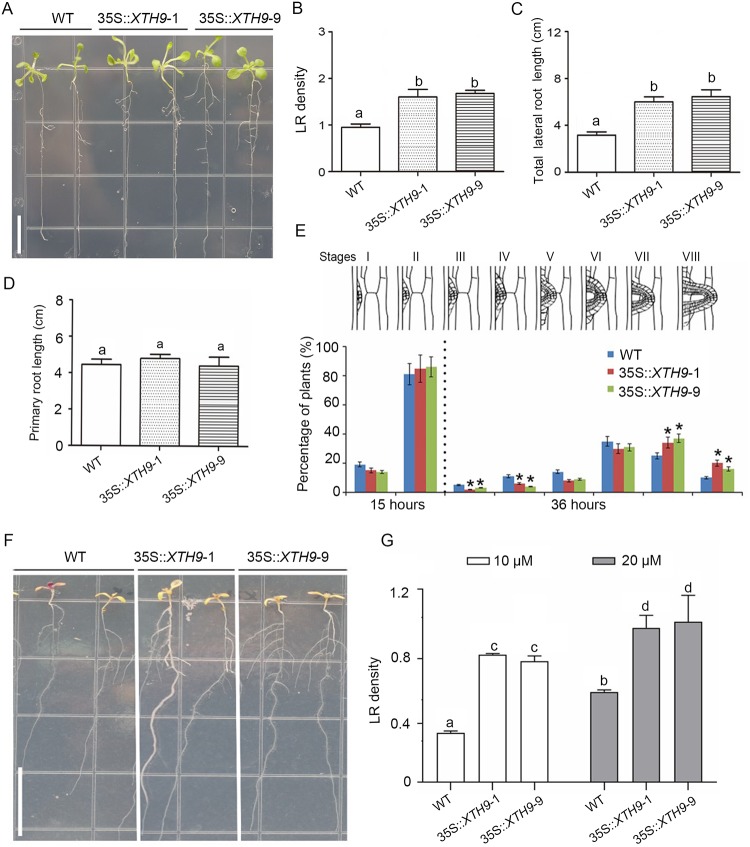
Overexpression of the *XTH9* gene increased lateral root (LR) development and improved low-nitrate tolerance. (A) Root phenotype observation and analysis of (B) LR density, (C) total LR length, and (D) primary root length in WT and *XTH9* overexpression lines. Bars with different capital letters indicate significant differences among different nitrate treatment in the given genotype by ANOVA analysis. The white bar indicates 1 cm. (E) Phenotypic analysis of lateral root emergence (LRE) was achieved by synchronizing LR formation with a gravity stimulus for 15 and 36 hours. Compared with the WT (Col-0) plants, the *XTH9* overexpression plants showed increased LRE. The data are shown as percentages, and the error bars represent SDs (n = 19–22). The error bars denote SDs (n = 16–23). Approximately 90 total LRP were observed in each plant. (F) WT and transgenic plants were grown vertically in media supplemented with 10 μM nitrate for 8 days. (G) Average LR lengths were quantified. The error bars represent SDs (n = 20–30). Bars with different capital letters indicate significant differences among different concentrations of nitrate treatment in the given genotype by ANOVA analysis. The white bars indicate 1.2 cm.

Due to the decreased LR growth in the *xth9* mutant, we investigated the root system development in constitutive *XTH9* overexpression lines. Nine of the 10 independent transgenic lines we obtained presented high *XTH9* expression levels (S6 Fig), and the transgenic plants exhibited substantially increased LRs (Fig 5A–5D). The WT and transgenic roots were then exposed to a gravitropic stimulus, and LRP were counted and staged at 15 and 36 hours pgi (Fig 5E). The WT plants accumulated stage I and II LRP at 15 hours pgi and stage III to VIII LRP at 36 hours pgi. LR initiation and first divisions were not affected in the 35S::*XTH9* plants, but showed an increased percentage of stage VII–VIII LRP at 36 hours pgi (Fig 5E). This result indicated that *XTH9* overexpression affected lateral root emergence (LRE). Because the *xth9* gene mutation decreased sensitivity to nitrate treatment, we investigated whether *XTH9* overexpression increased low-nitrate stress tolerance. The results showed that, on 10 μM nitrate media, WT and transgenic plants showed an N-deficient phenotype, but the transgenic plants exhibited much better growth and a substantially increased LR system (Fig 5F and 5G). Therefore, we concluded that *XTH9* overexpression improved low-nitrate tolerance.

### Auxin regulates *XTH9* expression in the roots via *ARF7* and *ARF19*

**Fig 6:**
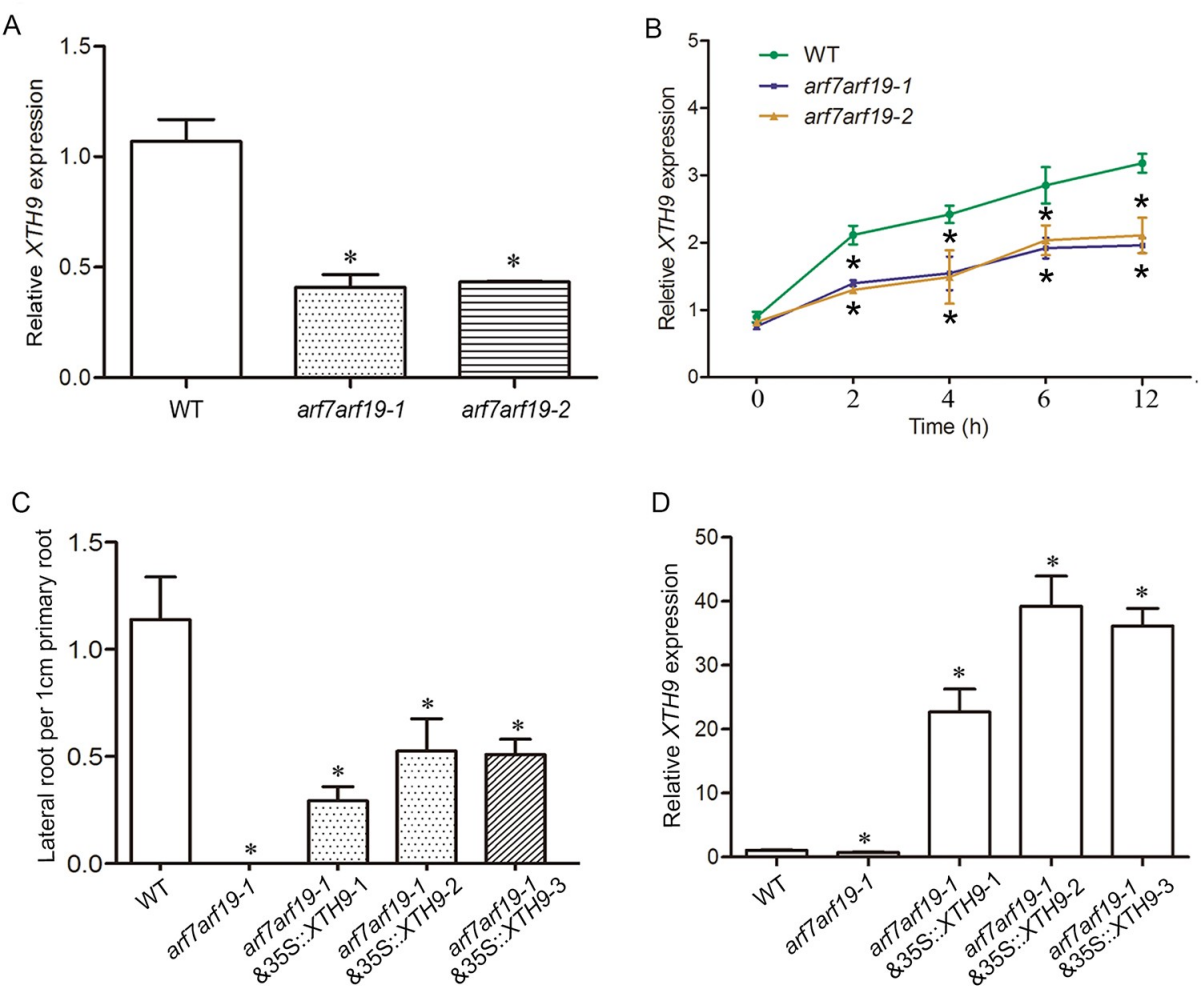
Auxin regulates *XTH9* expression in an *ARF7/19*-dependent manner. (A) *XTH9* expression level in the *arf7arf19* double mutant background. RNA was isolated from 7-day-old *Arabidopsis* roots. (B) After treatment with 1 mM IAA for the indicated time, the wild-type (WT) and *arf7arf19* double mutant roots were collected at the indicated time points. Values were determined from three independent plant roots. The auxin-dependent repression of *XTH9* expression in the roots is *ARF7/19* dependent. (C) Relative length of lateral root per 1 cm of primary root and (D) *XTH9* expression levels in the WT, *arf7arf19-1* and *arf7arf19-1* & 35S::*XTH9* transgenic plants. *indicates significant differences (*p*<0.05). The error bars represent SDs (n = 3).

The products of *ARF* genes function as TFs that regulate downstream auxin-responsive genes. Among these genes, *ARF7* and *ARF19* play critical roles during LR development and show redundant functions. The *arf7arf19* double mutant showed no LR initiation [50]; thus, we examined whether auxin regulates *XTH9* expression through *ARF7/19* in the roots. We initially investigated whether the regulatory mechanisms controlling auxin-promoted *XTH9* expression depend on ARF. We observed that the *XTH9* mRNA level was affected in the *arf7arf19* double mutant background (Fig 6A), suggesting that *XTH9* expression depends on the ARF TF [51–53]. Furthermore, transcript profiles of the roots of WT and *arf7arf19 Arabidopsis* mutants exposed to external auxin for varying lengths of time were examined by using RT-qPCR. Transcript profiling revealed that auxin-increased *XTH9* mRNA expression was affected in the *arf7arf19* mutant background (Fig 6B). Although *XTH9* showed sustained auxin-dependent induction, a weakened auxin effect was detected in the *arf7arf19* double mutants. Similarly, when the proXTH9::GUS reporter was expressed in *arf7arf19*, downregulated root GUS signaling was observed in *arf7/19* & proXTH9::GUS plants [22] (S7 Fig). Thus, we concluded that auxin regulates *XTH9* expression in an *ARF7/19*-dependent manner. To identify whether *XTH9* acts downstream of ARFs to regulate LR development, we introduced 35S::*XTH9* into *arf7arf19* mutants to get 35S::*XTH9* & *arf7arf19* plants. As with 35S::*XTH9*, the 35S::*XTH9* & *arf7arf19* plant showed more LR development, indicating *XTH9* is functional downstream of *ARF7/ARF19* (Fig 6C and 6D).

### Nitrate regulation of *XTH9* acts downstream of the AFB3-IAA14 signaling pathway

**Fig 7:**
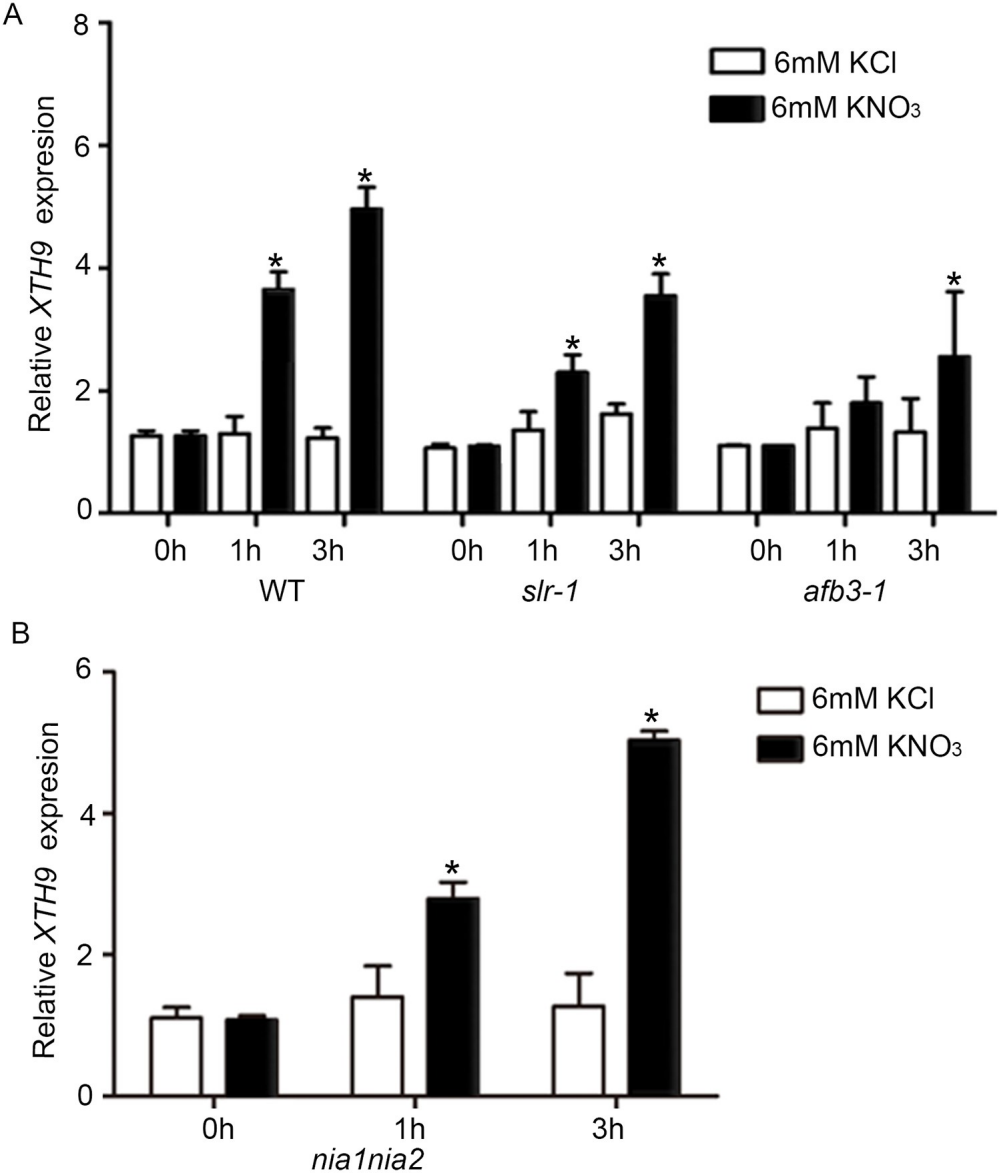
The nitrate response of *XTH9* is altered in *afb3-1*, *slr-1* mutant background and *XTH9* is still regulated by nitrate in NR-null *nia1nia2* mutant. (A) WT (Col-0) and *afb3-1* and *slr-1* mutant plants were grown on media supplemented with ammonium succinate for one week and subsequently treated with 6 mM KNO_3_ or 6 mM KCl for 1–3 hours. The *XTH9* gene expression level in the roots was measured via RT-qPCR. (B) Nitrate reductase-null plants (*nia1nia2*) were grown in media supplemented with ammonium succinate for one week and then treated with 6 mM KNO_3_ or 6 mM KCl for 1–3 hours. The RNA level of the *XTH9* gene was measured via RT-qPCR. KCl treatment results are shown with white bars, and the KNO_3_ treatment results are shown with black bars. *indicates significant differences (*p*<0.05). The error bars represent SDs (n = 18–24).

Previous studies involving systems approaches have revealed that the nitrate-regulated miR393/AFB3 module controls RSA [1, 2]. Because the cell wall-related gene *XTH9* is predicted to be involved in auxin and nitrate signaling, *XTH9* represents a likely downstream candidate of *AFB3* for mediating the effects of nitrate on LR development. Therefore, we assessed *XTH9* expression levels over time in response to nitrate treatment in WT, *afb3-1*, and *slr-1* (a gain-of-function mutation in the *SOLITARY-ROOT/IAA14* gene gain-of-function of IAA14) mutants using RT-qPCR. Compared with the WT plants, the mutant plants exhibited delayed transient responses to nitrate after treatment. The results showed that *AFB3-IAA14* (*SLR*) function is required for the nitrate-mediated regulation of *XTH9*. The nitrate responses of *XTH9* were compromised in the *afb3-1* and *slr-1* mutant backgrounds (Fig 7A). Previous studies have shown that AFB3 is induced by nitrate in a nitrate reductase-null *nia1nia2* double mutant. Given that *XTH9* function depends on *AFB3*, we assessed *XTH9* expression levels in the *nia1nia2* background in response to nitrate treatment. The results showed that these genes were still regulated by nitrate in the NR-null mutant, suggesting that the genes respond to nitrate, but not to N metabolites (Fig 7B), which is consistent with the hypothesis that the *XTH9* gene is regulated by nitrate signaling. In addition, the LR phenotype in nitrate-treated WT, *afb3-1*, and *slr-1* are shown in S8 Fig.

### The Dof TF *OBP4* negatively regulates *XTH9* in the roots

**Fig 8:**
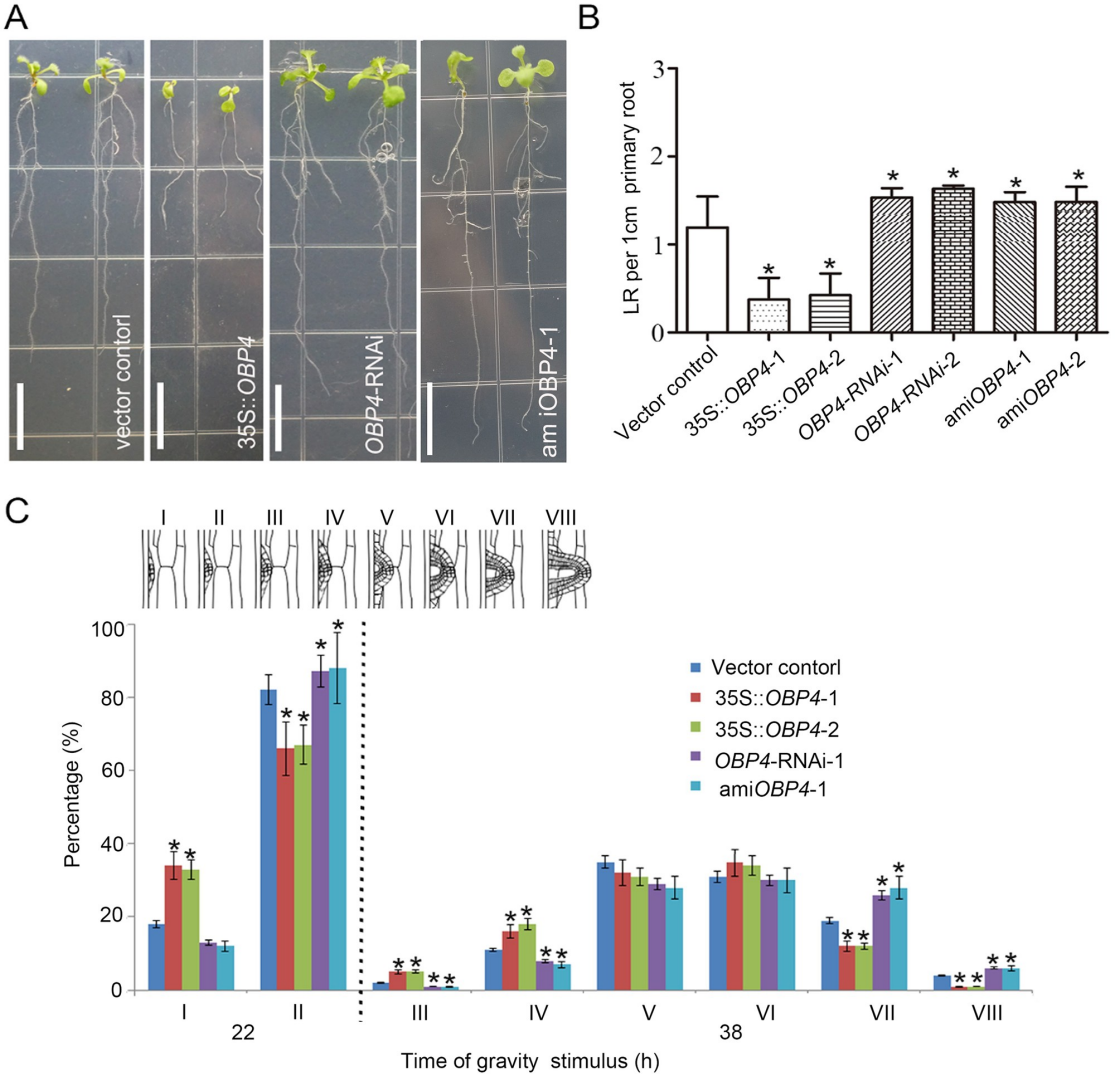
*OBP4* regulates plant root system architecture. (A) Phenotypic observation and (B) analysis of the root system in constitutive overexpression and knockdown *OBP4* transgenic plants. The white bar indicates 1.2 cm in length. (C) Phenotypic analysis of lateral root emergence (LRE) was performed by synchronizing lateral root (LR) formation with a gravity stimulus for 22 and 38 hours. Compared with that in the control plants, the LRE in the *OBP4* overexpression plants and *OBP4* knockdown plants was delayed and promoted, respectively. The data are shown as percentages and the error bars represent SDs (n = 8–12). Approximately 24–36 lateral root primordia (LRP) were observed in 35S::*OBP4* plants. Approximately 100 LRP were observed in *OBP4* knockdown transgenic plant. *indicates significant differences (*p*<0.05).

After assessing the *XTH9* promoter, we identified many Dof-binding motifs (Fig 8A). To independently assess the role of other regulatory regions, we used an *XTH9* promoter-deletion approach (S9A Fig). The approximately 2.3-kb-long *XTH9* promoter was truncated at four different positions (Δ1, Δ2, Δ3, and Δ4) to generate increasingly shorter promoter fragment lengths, after which these constructs were subsequently transformed into the *xth9* mutant background. Multiple transgenic lines expressing each proXTH9::*XTH9* promoter deletion were initially scored for complementation of the *xth9* LR phenotype. All Δ1 and Δ2 promoter deletion lines fully complemented the *xth9* LR defect (S9B Fig). This result demonstrates that the 1100-bp promoter sequence of *XTH9* is sufficient to promote LR emergence. In contrast, all Δ3 promoter deletion lines partially complemented the *xth9* LR phenotype, whereas no complementation was observed for any of the Δ4 promoter deletion lines (S9B Fig).

Based on our previous results, the *XTH9* expression level was downregulated in the Dof family TF *OBP4* overexpression transgenic lines, *OBP4* codes for a TF that negatively targets *XTH9* [54]. The *OBP4* expression pattern in the roots is similar to that of *XTH9* in both the LR and vascular tissue [55], and *OBP4* negatively regulated *XTH9* expression in the roots (S9C Fig). Similar to *XTH9*, *OBP4* also responds to nitrogen signals downstream of AFB3 [2, 12]. We performed ChIP analysis to test the relative enrichment of OBP4 in the selected fragments. An antibody directed against HA was used to precipitate OBP4::HA and its crosslinked DNA, which was isolated from pER8::OBP4::HA roots after estradiol induction. These results showed that OBP4 binded to *XTH9* gene promoter regions. Based on this information, we get the conclusion that *OBP4* negatively targets *XTH9* to participate in nitrogen signal regulation.

### *OBP4* negatively regulates plant root growth

To directly examine the functional importance of *OBP4* in *XTH9* nitrate-inducible expression, we identified mutants carrying a T-DNA insertion, namely, lines SALK_116433, SALK_118463, WiscDsLoxHs071, and CS69190. These mutations were unable to downregulate *OBP4* expression, most likely because all the T-DNA inserts were detected in the promoter region of *OBP4*. Studies have shown that overexpression of *OBP4* under a constitutive promoter resulted in dwarf plants with shorter primary roots and fewer LRs (Fig 8A and S10A Fig) [54, 55]. Furthermore, we generated *OBP4* knockdown lines by an RNA interference method (*OBP4*-RNAi) and artificial miRNA-mediated RNA silencing method (ami*OBP4*). Both *OBP4*-RNAi and ami*OBP4* transgenic plants significantly reduced the *OBP4* transcript level (S10B and S10C Fig). To validate the interference specificity, we quantified the levels of *OBP1*, the closest homolog of *OBP4*. *OBP1* transcripts were unaltered in *OBP4*-RNAi and ami*OBP4* seedlings (S10D Fig). In contrast to the 35S::*OBP4* plants, phenotype analysis showed that *OBP4*-RNAi and ami*OBP4* seedlings developed longer primary roots and more LRs than controls (Fig 8A and 8B). These results indicate that *OBP4* acts as a repressor of root growth. For 20 hours after gravity stimulation, the vector control root bends contained primarily stage I and stage II LRP, whereas, by 38 hours, many primordia were close to emergence (stage VI or VII). In the case of the 35S::*OBP4* plants, 20 hours after gravity stimulus, the transgenic plant roots proportionately displayed more stage I primordia than did the vector control roots. Thirty-eight hours after gravity stimulus, 35S::*OBP4* transgenic plants had a higher proportion of stage II to IV primordial, but the *OBP4*-RNAi and ami*OBP4* plants had a higher proportion of stage VII and VIII primordial (Fig 8C). These results were consistent with the hypothesis that *OBP4* functions in the regulatory pathway of LR emergence and indicate that *OBP4* negatively regulates LR growth.

### *XTH9* functions downstream of *OBP4* in control of LR growth in response to variation of nitrate concentrations

**Fig 9:**
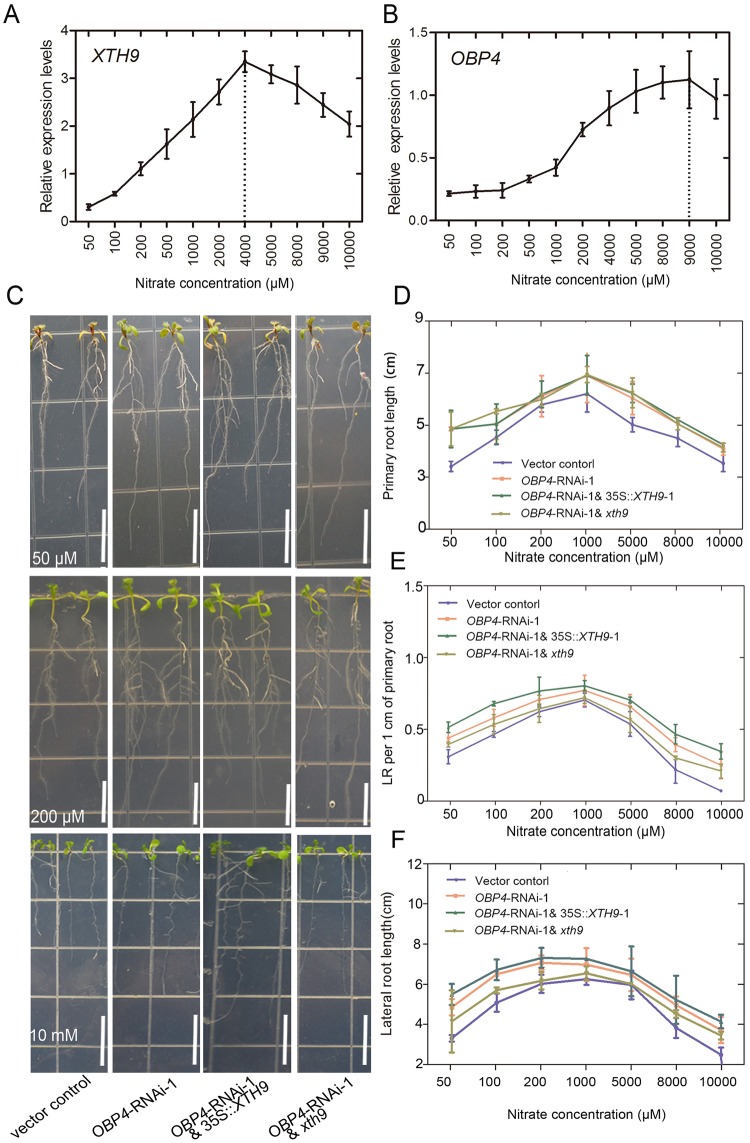
Relative expression of *XTH9* and *OBP4* genes in response to nitrate treatments and *OBP4*-*XTH9* in control of LR growth in response to variation of nitrate concentrations. Wild-type (WT) (Col-0) plants were grown on media supplemented with ammonium succinate for one week and subsequently treated with various concentrations of nitrate for 2 hours, and the relative (A) *XTH9* and (B) *OBP4* gene expression levels were assessed. (C) Phenotypic observations and analysis of (D) the primary roots, (E) the lateral root (LR) density, (F) the LR length of 4-day-old vector control, *OBP4*-RNAi, *OBP4*-RNAi-1 & 35S::*XTH9-1* and *OBP4*-RNAi-1 & *xth9* transgenic plants in response to various concentrations of nitrate (50, 100, 200, 1000, 5000, 8000, and 10000 μM) treatment for five days. LR density was calculated by dividing the LR number per 1 cm of primary root. *indicates significant differences (*p*<0.05). The error bars show SDs (n = 20–32). The white bars indicate 1.2 cm.

We tested how *OBP4* and *XTH9* regulated LR growth at nitrate concentrations ranging from 50 to 10000 μM (Fig 9A and 9B). First, we found that the expression of *XTH9* increased rapidly at low concentrations. The induction concentration of maximal *XTH9* expression was detected at 4000 μM. Higher nitrate concentrations inhibited *XTH9* expression (Fig 9A). However, the *OBP4* expression level was not increased under low nitrate concentrations of 50–1000 μM. Starting from nitrate induction with 1000 μM, the expression of *OBP4* increased rapidly. When the expression of *OBP4* reached the highest level, the nitrate-induced concentration was 8000–9000 μM (Fig 9B). The induction expression pattern indicates that *OBP4* may have a special role under high nitrate conditions.

In addition, we observed an altered nitrate response of *OBP4* in *afb3-1* and *slr-1* mutant backgrounds (S11 Fig). We further aimed to identify whether *OBP4* acted upstream of *XTH9* in controlling root development in response to nitrate. Because it is difficult to screen hygromycin-positive plants and 35S::*OBP4* plants are pollen sterile, we crossed the *OBP4*-inhibited transgenic plants with *xth9* mutants and 35S::*XTH9* plants to investigate how the coordination between *OBP4* and *XTH9* can regulate LR development under nitrate signaling. Phenotype analyses in vector control, *OBP4*-RNAi, *OBP4*-RNAi & 35S::*XTH9*, and *OBP4*-RNAi & *xth9* were performed (Fig 9C). The results showed that *OBP4*-RNAi had more LRs and longer primary roots than the vector control (Fig 9D). *OBP4*-RNAi & 35S::*XTH9* further increased the LRs, and *OBP4*-RNAi & *xth9* decreased LRs more than *OBP4*-RNAi plants (Fig 9E and 9F). However, the lengths of primary roots in *OBP4*-RNAi, *OBP4*-RNAi & 35S::*XTH9*, and *OBP4*-RNAi & *xth9* plants were almost identical, but longer than the vector control (Fig 9D). The results showed clearly that *OBP4* negatively regulates root growth, while *XTH9* functions downstream of *OBP4* and promotes LR growth.

**Fig 10:**
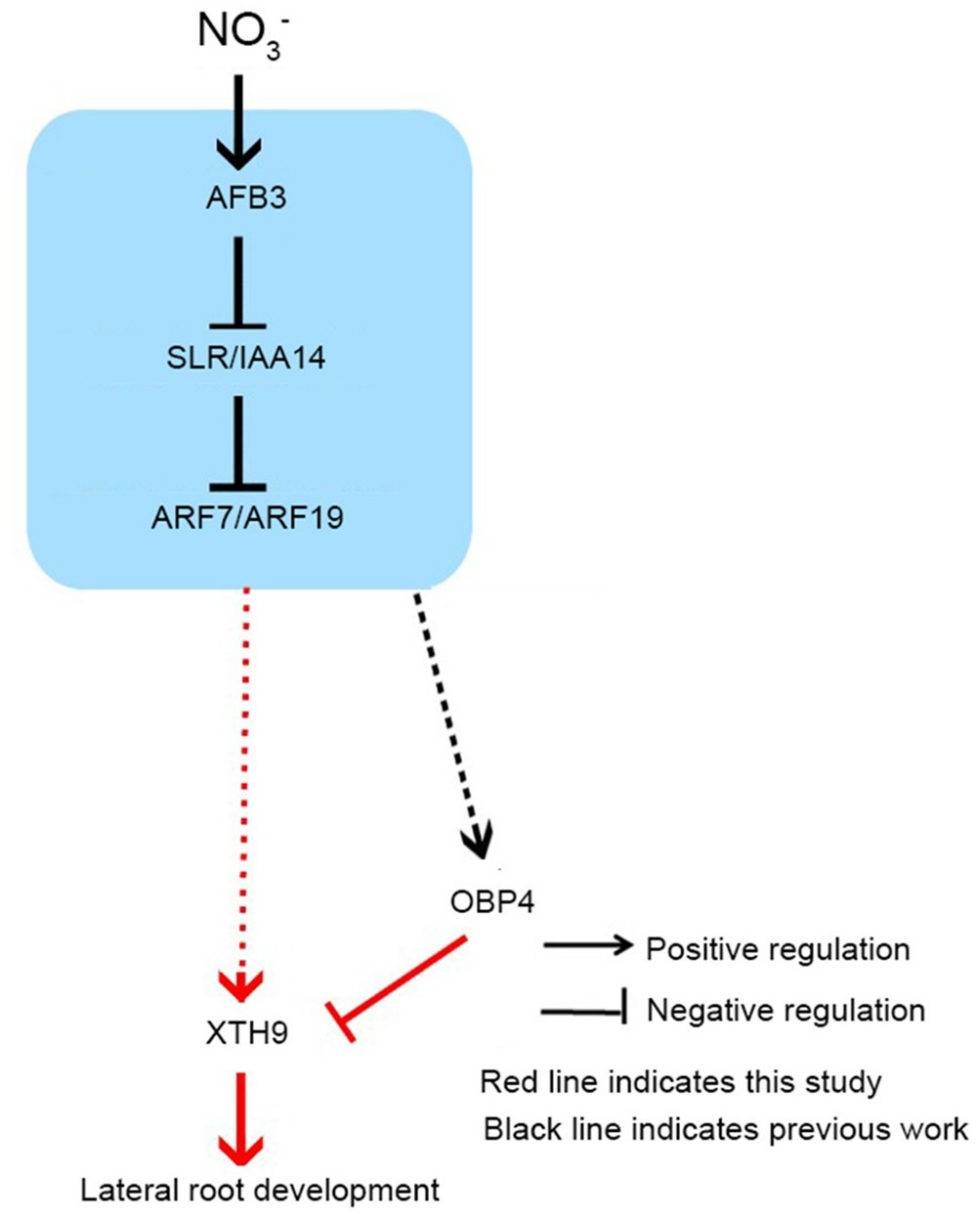
The *OBP4*-*XTH9* module regulates lateral root (LR) development in response to nitrate signaling. Temporal control of the lateral root emergence (LRE) gene regulatory network involving *OBP4* and *XTH9*. Auxin is perceived by AFB3 and triggers the degradation of IAA14, which releases ARF7/19. Auxin regulates *XTH9* expression in an ARF7/19-dependent manner. *XTH9* is also negatively regulated by *OBP4*, which fine-tunes the *XTH9* expression level in response to environmental nitrate availability. This synergistic regulation of *OBP4*-*XTH9* has a specific function in lateral root (LR) development in response to nitrate signaling in *Arabidopsis*.

We also treated the vector control, *OBP4*-RNAi, *OBP4*-RNAi & 35S::*XTH9*, and *OBP4*-RNAi & *xth9* plants with different concentrations of nitrate. We observed that the growth of LRs was nitrate-dependent (Fig 9C). In the lower concentration range (50–1000 μM), the expression of the *XTH9* gene upregulated rapidly and the expression of the *OBP4* gene upregulated slowly with an increase of nitrate concentration. *XTH9* promoted the growth of LRs induced by low nitrate concentration (Fig 9A). It was also observed that *XTH9* played an important role in promoting LR growth in low concentrations. However, the expression of *OBP4* was increased at higher concentrations (Fig 9B). *OBP4* plays an important role in restraining the growth of LRs. Therefore, the growth of LRs in vector control plants decreased most rapidly with an increase of nitrate at a range of high concentrations (Fig 9E and 9F). Altogether, our current model demonstrated that under low nitrate conditions, *XTH9* mainly worked downstream of *ARF7/9* to promote LR growth. However, under high nitrate conditions, *OBP4* antagonistically regulated *XTH9* at the late stage of nitrogen stimulation, which inhibited *XTH9* expression level to fine-tune LR maintenance. Therefore, in response to signaling at various concentrations of nitrate the *OBP4-XTH9* regulatory module elaborately controls LR growth in *Arabidopsis* (Fig 10).

## Discussion

In the *Arabidopsis* genome, 33 genes encoding XTH proteins have been identified. Individual members of this gene family exhibit specific expression patterns, temporally, and spatially [38]. Here, we isolated 11 of these genes, namely, *XTH1–11*, which are clustered into group I (S12 Fig). First, we determined whether these genes are involved in primary and/or lateral root development. The results from 90° gravitropic stimulus assays revealed that LR initiation occurs in a highly synchronized manner at the outer surface of bending roots. We profiled the class 1 *XTH* genes at all stages of LR development, and the results showed that *XTH9* mRNA levels were highly upregulated during LR development (Fig 1B and 1C). We subsequently isolated mutants of each *XTH* gene family member. We carefully observed the effects of *XTH9* on LR development and compared it with that in the WT plants. The density of emerged LRs at stages VII and VIII decreased in the *xth9* mutant plants (Fig 2G). Although *xth9* mutant showed decreased LR growth under all nitrate concentrations, with the increase of nitrogen concentration, the curve curvature of lateral root density of mutant was significantly lower than that of control group. And in the *XTH9* over-expressing lines, the variation range of lateral roots treated with exogenous nitrogen was smaller than that of the wild type. So, we got the conclusion that *XTH9* played an important role in mediating plant lateral root development in response to exogenous nitrogen signals. Furthermore, we generated transgenic proXTH9::GUS lines and observed GUS signals during the formation of LRP and during LR initiation and elongation (Fig 3A–3H). The results of transcript profiling and reporter studies showed that *XTH9* was expressed in response to nitrate treatment (Fig 3I–3M). In the *xth9* mutant, the sensitivity to nitrate-promoted LR growth was defective, but the main root elongation was not affected (Fig 4G). Moreover, in response to increasing nitrate concentrations, the average LR length curve for the *xth9* mutant plants was lower than that for Col-0 plants (Fig 4H). In addition, *XTH9* gene overexpression increased in lateral roots and enhanced tolerance to low nitrate stress (Fig 5).

Previous systems approaches have revealed that the nitrate-regulated miR393/AFB3 module controls RSA [2]. To identify whether the nitrogen-responsive cell wall-related *XTH9* gene controls root development downstream of *AFB3*, we determined the expression levels of *XTH9* over time in response to nitrate treatment in WT and *afb3-1* and *slr-1* mutants. The nitrate response of *XTH9* was clearly compromised in the mutant background (Fig 7A). We also observed that *XTH9* was still regulated by nitrate in the NR-null mutant (Fig 7B), suggesting that this gene responds to nitrate signaling, but not to N metabolites. The nitrate-specific induction of *AFB3* in the roots might control a specific component of Aux/IAA and ARF TFs that facilitate LR growth. In *Arabidopsis*, LR development depends on multiple Aux/IAA-ARF modules to generate new LRs. The IAA14 (SLR)-ARF7 and ARF19 modules regulate the posterior asymmetric cell division of founder cells in preparation of LR initiation. We initially investigated whether these ARF-dependent regulatory mechanisms control auxin-promoted *XTH9* expression. We observed that the *XTH9* mRNA level was clearly downregulated in the *arf7arf19* mutant background (Fig 6A). Furthermore, the 35S::*XTH9* & *arf7arf19* plant showed more LR development than the *arf7arf19* plant, indicating *XTH9* is functional downstream of *ARF7/ARF19* (Fig 6C and 6D). In summary, the present study identified the *XTH9* components that are downstream of the AFB3-dependent network and that regulate root system growth in response to nitrate.

To independently assess the role of other regulatory regions, we used an *XTH9* promoter-deletion approach. The 2.3-kb-long *XTH9* promoter was truncated at four different positions to generate increasingly shorter promoter lengths, and the resulting constructs were subsequently transformed into the *xth9* mutant background. All Δ1 and Δ2 promoter deletion lines fully complemented the *xth9* LR defect (S9A and S9B Fig). This result demonstrates that the 1100-bp upstream promoter sequence of *XTH9* is sufficient to drive nitrate-inducible expression and promote LR emergence. Therefore, we concluded that the Dof-binding motifs of the *XTH9* gene promoter are important for gene activity. In addition, OBP4 negatively regulates *XTH9* expression in the roots (S9C Fig). The *OBP4* expression pattern in the roots is similar to that of *XTH9* in both LRs and vascular tissue [54, 55]. OBP4 is a promising candidate as an intermediary transcriptional regulator of *XTH9*. Therefore, we subsequently used constitutive *OBP4* overexpression and *OBP4* knockdown lines. In the LR system, 35S::*OBP4* inhibited LR development, while the *OBP4*::RNAi and pER8::ami*OBP4* plants promoted LR development (Fig 8A and 8B). Furthermore, by treating the vector control, *OBP4*-RNAi, *OBP4*-RNAi & 35S::*XTH9* and *OBP4*-RNAi & *xth9* with various concentrations of nitrate, we found that *OBP4*-RNAi plants increased LR growth under high nitrate conditions (Fig 9). We concluded that *OBP4* is involved in the nitrate signaling pathway.

Here, we identified a nitrogen-responsive signaling module comprising the cell wall-related gene, *XTH9*, and the Dof TF, OBP4, downstream of *AFB3*. This module plays an important role in controlling root development. We proposed that the OBP4 TF is involved in the regulation of LRE and the LR elongation stage. However, the major role of *XTH9* seems to be associated with LR elongation. Although *OBP4* can negatively regulate *XTH9* expression level, it may associate with other downstream target genes involved in the nitrate signaling pathway. Moreover, phenotype analyses in vector control, *OBP4*-RNAi, *OBP4*-RNAi & 35S::*XTH9*, and *OBP4*-RNAi & *xth9* transgenic plants showed that *OBP4* and *XTH9* respond to changes in nitrate concentration in plant LR growth (Fig 9). Altogether, we propose that under low nitrate conditions, *XTH9* mainly works downstream of *ARF7/9* to promote LR growth, while under high nitrate conditions, *OBP4* negatively regulates *XTH9*, which inhibits *XTH9* expression level and results in the further inhibition of LR growth. Therefore, *OBP4* and *XTH9* function as a regulatory module in response to various concentrations of nitrate signaling to control LR growth in *Arabidopsis* (Fig 10).

In fact, we propose that both the expression of *XTH9* and *OBP4* are regulated by nitrogen signals through *ARF7/19*, but their functions are antagonistic: *XTH9* positively controls LR development while *OBP4* negatively controls LR development via suppressing *XTH9*. *OBP4* is a safeguard mechanism that prevents the over-accumulation of XTH9 in response to nitrogen signals. It is possible that *XTH9* is strongly induced for LR establishment at the beginning of nitrogen stimulation, once XTH9 reaches a saturated level; the plant initiates a feedback mechanism to suppress *XTH9* expression. It is also reasonable to assume that plants do not need too much XTH9 once adequate levels are reached in response to nitrogen signals, thus the plant initiates the expression of *OBP4* to suppress the transcription of *XTH9* during the later stage. As a result, the plant fine-tunes the number of LR by an elaborate *OBP4-XTH9* regulatory module, including *OBP4*, which antagonistically suppresses the expression of *XTH9* to coordinate the activity of *XTH9* and LR architecture. A mechanism in plants to regulate LR growth in response to different concentrations of nitrate in the surrounding environment is beneficial in the effective utilization of nitrogen by plants.

## Materials and methods

### Material and plant growth conditions

The Col-0 ecotype of *Arabidopsis thaliana* (L.) Heynh was used in all experiments. The germinated seeds were transferred to media in growth chambers at 22±2°C under a 16-hour/8-hour (day/night) photoperiod, and the seeds were surface sterilized [56]. The seeds were then subjected to synchronized germination at 4°C in the dark for 3 days, after which they were subsequently transplanted to Murashige and Skoog (MS) media under a 16-hour/8-hour (day/night) photoperiod. The *arf7/arf19-1* and *arf7/arf19-2* mutants [51], *xth9* mutants (SALK_101024, SALK_063401, SALK_023274, SALK_002571), *nia1nia2* double mutant (CS2356) and *obp4* (SALK_116433, SALK_118463, WiscDsLoxHs071, CS69190) mutants were obtained from the Arabidopsis Biological Resource Center.

### Plasmid construction and transgenic plant generation

*XTH9* cDNA was cloned from *Arabidopsis thaliana*. The *XTH9* gene was then subcloned into a pHB vector to generate 35S::*XTH9* transgenic lines. The 2.3-kb-long *XTH9* promoter was fused with the *XTH9* coding DNA sequence (CDS) and then subcloned into a pCAMBIA1300 vector. Generation of transgenic *xth9* mutants expressing the *XTH9* chimeric gene was carried out as previously described [57, 58]. For inducible and constitutive *OBP4* overexpression, the *OBP4* ORF was cloned into the binary vector pER8 and pHB. For inducible and constitutive knockdown of *OBP4*, about 250 bp target sequence was cloned from genome. This sequence was introduced into the pCAMBIA1301 vector in the sense and antisense orientation. Next, the entire fragment was removed by digesting the pCAMBIA1301-based construct, and the fragment was inserted into pHB vector. We obtained several *OBP4* knockdown transgenic lines, the highly downregulated lines pHB::*OBP4*::RNAi were used for our experiments. For artificial microRNA (amiRNA) mediated gene silencing of *OBP4*. We used the WMD3 online tool (http://wmd3.weigelworld.org) to the design of amiRNA specific to *OBP4* coding region, the artificial miRNA vector pRS300 was requested from the Weigel lab. The final fragment was inserted into pHB vector. We obtained several transgenic lines, the highly *OBP4* downregulated lines pHB::ami*OBP4* was used for our experiments. Col-0 plants were transformed by the floral-dip method. The positive transgenic plants were screened by 50 mg/L Hygromycin (Roche, USA), and RT-qPCR was performed to measure the gene expression levels in the transgenic plants; the primers used are listed in S1 Table in Supporting Information.

### Cloning of full promoters and promoter deletions

Point-mutated promoters were cloned back into pCAMBIA1300-XTH9-GUS and sequenced to ensure that no other mutations were generated during PCR. For the PCR-generated promoter deletions, a combination of primers for Δ1, Δ2, Δ3, and Δ4 was used. The purified PCR products were subsequently subcloned into the pCAMBIA1300 vector.

### Expression analysis via RT-qPCR

Total RNA was isolated using an RNeasy Plant Mini Kit (Qiagen, USA) and stored at -80°C. One gram of total RNA was used for cDNA synthesis via Novo Script First-Strand cDNA Synthesis SuperMix E041 (Novoprotein, China). The experiments were conducted using Hieff qPCR SYBR Green Master Mix (Yeasen, China) according to the manufacturer’s instructions. The data were analyzed by using LightCycler 96 analysis software 1.1 (ΔΔC_T_ method). The *AtACT2* gene was used as an internal control. The RT-qPCR primers used are listed in S2 Table, and the assays were performed in triplicate.

### Nitrate treatment assays

Approximately 1000 seedlings were hydroponically planted on nitrogen-free basal salt media (M531, Phytotechnology Laboratories) that were supplemented with various concentrations of nitrate (10, 50, 500, 1000, 5000 and 10000 μM) and 1% sucrose. The seedlings were then subjected to a 16-hour/8-hour (day/night) photoperiod at 22°C.

### Histochemical analysis and microscopy

With respect to the histochemical analysis of GUS activity, *Arabidopsis* seedlings were incubated at 37°C in reaction buffer (100 mM sodium phosphate buffer [pH 7.0], 0.5 mM potassium ferricyanide, 0.5 mM potassium ferrocyanide, 0.1% [vol/vol] Triton X-100 and 0.1% [wt/vol] sodium lauroyl sarcosine) plus 1 mM X-Glu. GUS staining and clearing was performed as previously described [54]. The seedlings were cleared and imaged using differential interference contrast (DIC) optics via a Leica microscope, and at least 12 plants were used for each treatment. The activity of β-glucuronidase (GUS) in intact plant tissue was measured using 4-methylumbelliferyl β-D-glucuronide (4-MUG) as a substrate according to the methods of [59].

### Analysis of root architecture traits

Initiating and emerging LRs (stages I, II, III, IV, V, VI, VII and VII) [22] were counted using DIC optics via a Leica microscope. For root measurements, the plants were scanned using a Nikon photo scanner, and the roots were measured using ImageJ software. The data were subsequently analyzed using Graph Pad Prism 5 software.

### XET activity assay

The XET activity of XTH was determined according to a method reported by Vissenberg [60]. The roots were cultured in a 5 mM XGO-SR mixture (XLLG-SR.XXLGSR.XXXG-SR) dissolved in 25 mm MES buffer at a pH of 5.5 for 1 hour and then rinsed with ethanol: formic acid: water (15:1:4, v/v/v) for 10 minutes to remove the remaining unreacted residue XGO-SR. The XGO-SR was further incubated overnight in 5% formic acid to remove exogenous, non-wall-bound XGO-SR [61]. Samples were mounted on glass slides and inspected under a confocal microscope using excitation light of 540 nm.

### Root cell wall extraction

Extraction of root crude cell wall materials were performed according to Zhu [37] with minor modifications. Roots were ground with a mortar and pestle in liquid nitrogen and then homogenized with 75% ethanol for 20 min in an ice-cold water bath. The sample was then centrifuged at 8000 rpm for 10 min, and the supernatant was removed. The pellets were homogenized and washed with acetone, methanol:chloroform at a ratio of 1:1, and methanol, respectively, for 20 min each, with each supernatant being removed after centrifugation between the washes. The remaining the cell wall material was dried and stored at 4°C for further use.

### Determination of total sugar residues

The content of total sugar residues in the hemicellulosic fractions was determined by the phenol-sulfuric acid method and expressed as Glc equivalents. Briefly, 200 mL of hemicellulose extracts was incubated with 1 mL of 98% H_2_SO_4_ and 10 mL of 80% phenol at room temperature for 15 min and then incubated at 100°Cfor 15 min. After cooling, the absorbance at 490 nm was measured spectrophotometrically.

### MALDI-TOF mass spectrometry analysis of XyG oligosaccharides

Arabidopsis roots were preserved in 100% ethanol. At 37°C overnight, 1 unit of xyloglucanase (Megazyme, Brae, Ireland) was treated with 50 mM sodium acetate buffer (pH 5.0) to remove ethanol and rehydrated to produce xylooligosaccharide. MALDI-TOF mass spectrometry of XyG oligosaccharides was recorded with an Applied Biosystems using super-DHB (Sigma-Aldrich, USA) as a matrix [62, 63].

### Chromatin immunoprecipitation (ChIP) assay

The ChIP assays were performed as described previously with some changes [54]. In brief, 5 g of 8-day-old pER8::*OBP4*-HA transgenic seedlings were extracted and immersed in 1% formaldehyde to cross-link DNA with DNA-binding proteins. Next, the chromatin pellets were extracted and sheared by sonication. Anti-HA antibody was used to immunoprecipitate the DNA-OBP4 protein complexes. DNA was released by protease K and purified for PCR analysis. The enrichment of DNA fragments was checked by qRT-PCR. Primers are indicated in S2 Table.

### Root phenotyping

The seedlings were grown on vertical plates subjected to 90° gravity stimulation for 54 hours [45]. In addition, 10-day-old seedlings were grown vertically and harvested to analyze the developmental stages of LRP. In this phenotypic study, the total number and stages of LRP were counted and determined according to the methods of Malamy and Benfey [64, 65]. The root length was measured using ImageJ software.

### Statistical analysis and multiple alignments

Statistics were performed with Graphpad 8.0, by using Student’s *t*-test or ANOVA analyses. Differences were considered significant when *p*<0.05. Multiple alignments of the predicted amino acid sequences and phylogenetic analyses were performed using DNAMAN 6.0 and MEGA 4.1 software, respectively [66].

### Accession numbers

The *Arabidopsis* Genome Initiative locus identifiers for the genes mentioned in this article are as follows: *XTH9*, At4g03210; *ARF7*, At5g20730; *ARF19*, At1g19220; *OBP4*, At5g60850; *NIA1*, At1g7760; *NIA2*, At1g37130; *IAA14*, At4g14550; and *AFB3*, At1g12820.

## Supporting information

S1 Fig *LBD29* expression pattern during LR initiation. *LBD29* expression pattern at each time point during LR initiation every 6 hours from 6 to 54 hours pgi. The bending roots of a population of 5-d-old seedlings were microdissected at each of the 10 time points and used for RNA extraction (approximately 200 per time point). The error bars show the SDs (n = 3).
(TIF)

S2 Fig RT-PCR analysis of *XTH* genes expression level in WT and mutants and root phenotype analysis of *xth4* and *xth10* mutants. (A) RT-PCR analysis of *XTH* genes expression level in WT and T-DNA insertion mutants. Root phenotypes of WT plants and the *xth4*, *xth10* mutants. The plants were grown vertically on media for 10 days. (B) Primary root. (C) LR density analysis of WT plants and the mutant lines. The error bars denote the SDs. *Actin2* was used as internal control.
(TIF)

S3 Fig *XTH9* mutation affects cell wall properties, *XTH9* expression patterns and it’s Contribute to in vivo XET activity. (A) Total sugar residues in extractable hemicellulose of Col-0 and *xth9* mutant. Cell wall material from roots was fractionated into different polysaccharide classes. Data are means ± SD. n = 3 (B) Cell wall material was extracted from Col-0 and *xth9* mutant roots and digested with XEG. The oligosaccharides obtained were analyzed by MALDI-TOF MS. Data are means ± SD; n = 2. The asterisk shows a significant difference between *xth9* and Col-0 at *p* < 0.05 by Student’s t test. (C) Analysis of the *XTH9* gene expression patterns in seedling, root, shoot, rosette leaf, flower, and bud tissues. The error bars show the SDs (n = 6). The asterisk (*) shows a significant difference at *p*<0.05 by Student’s *t* test. (D) XET activity action expressed as fluorescence relative to untreated wild type. Roots were subjected to cytochemical assays of XET action for 1 h. Data are means SD (n = 3). (*) indicate significant differences at *p* < 0.05 by Student’s *t* test.
(TIF)

S4 Fig Relative *XTH9* expression levels after various treatments. Ten-day-old wild-type plants grown on half-strength MS-agar plates were treated with 200 mM mannitol, heat (30°C), 100 mM NaCl, 500 μM KNO_3_, 1 mM KH_2_PO_4_ and plant growth hormones (1 μM ABA and 20 μM GA) for 3 and 8 hours. For drought treatment, the plants were transferred to dry 3M paper for 3 and 8 hours. RT-qPCR was used to check *XTH9* expression levels at various time points. The error bars show the SDs (n = 3).
(TIF)

S5 Fig Analysis of *xth9* mutant complementary lines in response to nitrate treatments. LR density (number of LRs per 1 cm of primary root length) in WT and complimentary lines grown in media supplemented with various concentrations of nitrate. The error bars show the SDs (n = 3).
(TIF)

S6 Fig Analysis of the 35S::*XTH9* transgenic plants. ***XTH9* expression level in the 2-week-old 35S::*XTH9* transgenic plant leaves.** *indicates significant differences (p<0.05). The error bars show the SDs (n = 3).
(TIF)

S7 Fig Analysis of relative proXTH9::GUS activity in the 7-day-old WT and *arf7arf19* double mutant roots. *indicates significant differences (p<0.05), and the error bars show the SD (n = 3).
(TIF)

S8 Fig LR phenotype of nitrate-treated WT, *afb3-1* and *slr-1* plants. (A) Observations of wild-type, *afb3-1* and *slr-1* plant root development in response of 500 mM nitrate treatment for 2 day. The bar indicates 1 cm. (B) Analysis of LR density (LR per 1 centimeter of primary root). *indicates significant differences (*p*<0.05), and the error bars show the SD (n = 3).
(TIF)

S9 Fig Identification of the *XTH9* upstream Dof transcriptional regulator OBP4. (A) The PLACEcare online tool was used to search for motifs. Many motifs including GATA-box-binding elements, the W-box elements and Dof TF-binding elements were found in the *XTH9* promoter. Representation of the *XTH9* full promoter from -2314 bp to the start codon (ATG). Promoter deletions (named Δ1, Δ2, Δ3 and Δ4) were generated and cloned upstream of *XTH9*::GUS. Dof-binding motifs are indicated as vertical solid lines. The sequence areas used for the ChIP experiment are marked in the gene promoters (from a to f, top panel). The number of Dof-binding motifs is indicated for each promoter deletion below the panel. (B) LR density measurements (number of LR per 1 cm of primary root length). The error bars represent the SDs. (n≥10). (C) Relative *XTH9* activity in pER8 vector transgenic lines and inducible *OBP4* expression and RNAi lines before and after 20 μM estradiol induction for 2 days. *indicates significant differences (*p*<0.05), and the error bars show the SD, n = 3. (D) 8-day-old pER8::OBP4::HA transgenic plants grown on MS-agar plates were used for ChIP assays. The enrichment shown was calculated as the DNA level of each fragment in the β-estradiol-treated sample divided by that in the DMSO-treated sample. Anti-HA antibody was used to precipitate OBP4-HA. Three measurements were averaged for individual assays. Bars indicate the SD. The values in Col-0 plants were set to 1 after normalization to ACT2 for qPCR analysis. Asterisks indicate significant differences, *p*< 0.05.
(TIF)

S10 Fig *OBP4* expression in 35S::*OBP4*, RNAi-*OBP4* and ami*OBP4* transgenic plants. (A-C) Relative *OBP4* expression levels in the 2-week-old 35S::*XTH9*, RNAi-*OBP4* and ami*OBP4* transgenic plants leaves. (D) *OBP1* expression levels in the vector control and RNAi-*OBP4*-1 and ami*OBP4*-1 transgenic plants. *indicates significant differences (*p*<0.05). The error bars show the SDs (n = 3).
(TIF)

S11 Fig The nitrate response of *OBP4* in the *afb3-1* and *slr-1* mutants. WT (Col-0), *afb3-1* and *slr-1* mutant plants were grown in media supplemented with ammonium succinate for one week and subsequently treated with 5 mM KNO_3_ or 5 mM KCl for 1–3 hours. The *OBP4* gene expression level in plant roots was measured via RT-qPCR. The KCl treatment results are shown with white bars, and the KNO_3_ treatment results are shown with black bars. *indicates significant differences (*p*<0.05), and the error bars show the SDs (n = 12–18).
(TIF)

S12 Fig Multiple sequence alignment and phylogenetic analysis of class 1 *XTH* genes. (A) Alignment and (B) Phylogram of *Arabidopsis* class 1 XTH family proteins. Multiple sequence alignment of the predicted amino acid sequence and phylogenetic analysis were performed via DNAMAN 6.0 and MEGA 4.1 software.
(TIF)

S1 Table Primers used for plasmid construction and mutant isolation. (DOCX)

S2 Table Gene-specific primers used in the qPCR experiments. (DOCX)

S3 Table T-DNA insertion lines of class 1 *XTH* genes. (DOCX)
