# Supplementary figures and images for: Nitrate-responsive OBP4-XTH9 regulatory module controls lateral root development in Arabidopsis thaliana
Source: PLoS Genet. 2019 Oct 18;15(10):e1008465. doi: 10.1371/journal.pgen.1008465 (PMC6821136; doi:10.1371/journal.pgen.1008465)

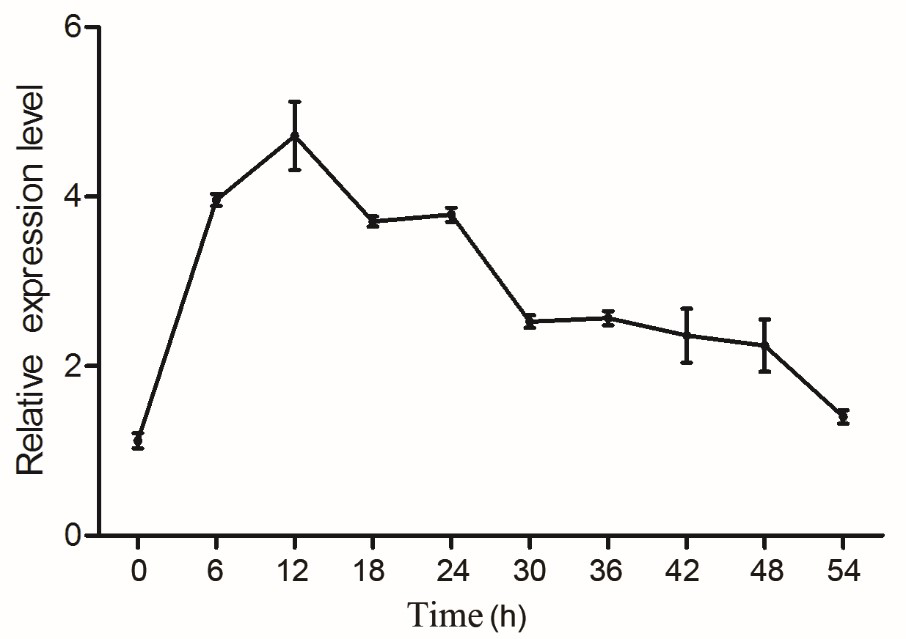

Supplement: S1 Fig — LBD29 expression pattern at each time point during LR initiation every 6 hours from 6 to 54 hours pgi. The bending roots of a population of 5-d-old seedlings were microdissected at each of the 10 time points and used for RNA extraction (approximately 200 per time point). The error bars show the SDs (n = 3). (TIF) [file pgen.1008465.s001.tif]

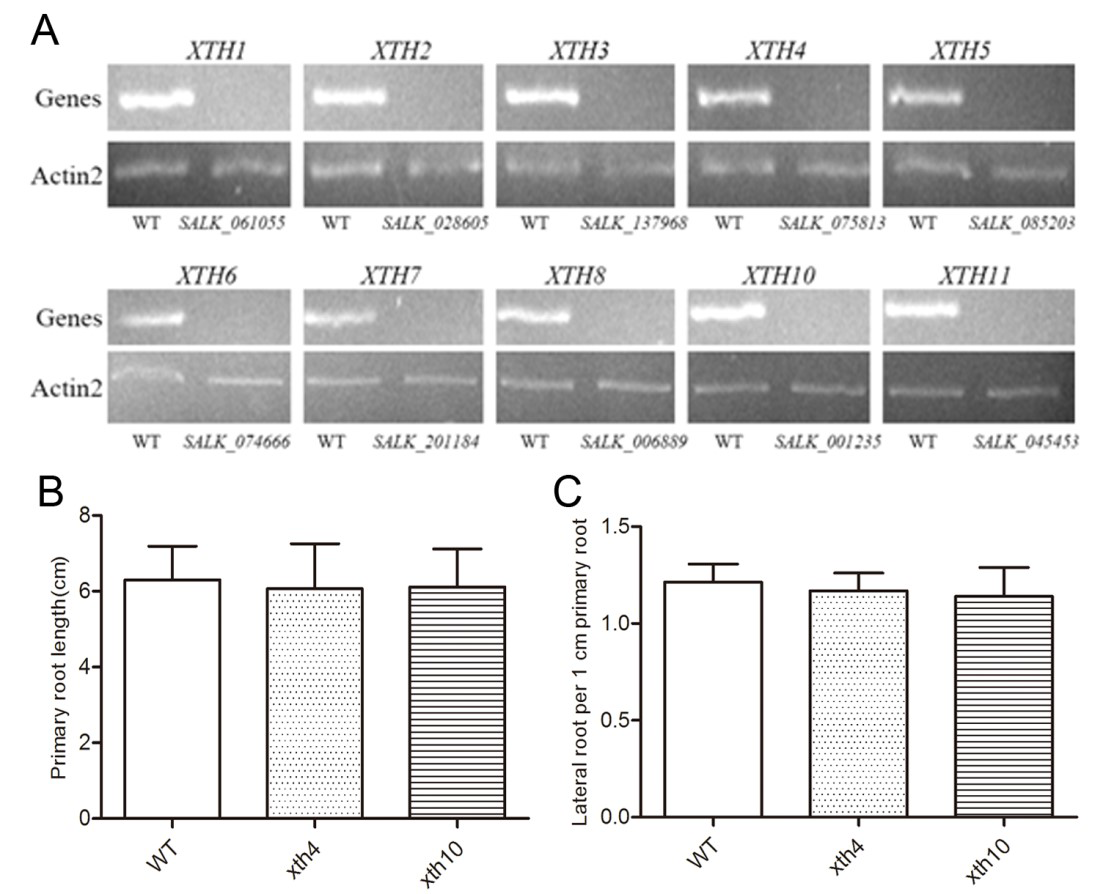

Supplement: S2 Fig — (A) RT-PCR analysis of XTH genes expression level in WT and T-DNA insertion mutants. Root phenotypes of WT plants and the xth4, xth10 mutants. The plants were grown vertically on media for 10 days. (B) Primary root. (C) LR density analysis of WT plants and the mutant lines. The error bars denote the SDs. Actin2 was used as internal control. (TIF) [file pgen.1008465.s002.tif]

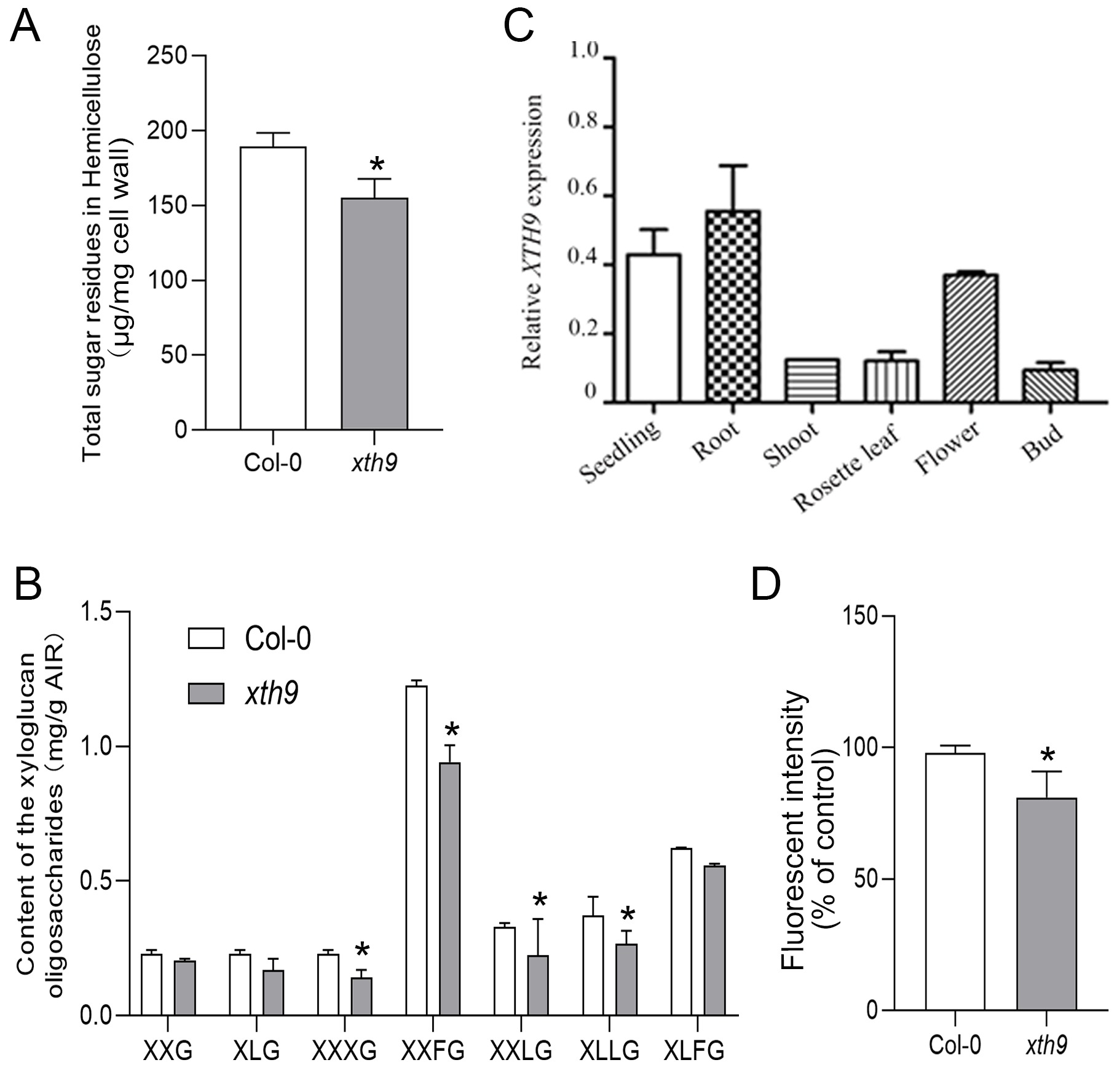

Supplement: S3 Fig — (A) Total sugar residues in extractable hemicellulose of Col-0 and xth9 mutant. Cell wall material from roots was fractionated into different polysaccharide classes. Data are means ± SD. n = 3 (B) Cell wall material was extracted from Col-0 and xth9 mutant roots and digested with XEG. The oligosaccharides obtained were analyzed by MALDI-TOF MS. Data are means ± SD; n = 2. The asterisk shows a significant difference between xth9 and Col-0 at p < 0.05 by Student’s t test. (C) Analysis of the XTH9 gene expression patterns in seedling, root, shoot, rosette leaf, flower, and bud tissues. The error bars show the SDs (n = 6). The asterisk (*) shows a significant difference at p<0.05 by Student’s t test. (D) XET activity action expressed as fluorescence relative to untreated wild type. Roots were subjected to cytochemical assays of XET action for 1 h. Data are means SD (n = 3). (*) indicate significant differences at p < 0.05 by Student’s t test. (TIF) [file pgen.1008465.s003.tif]

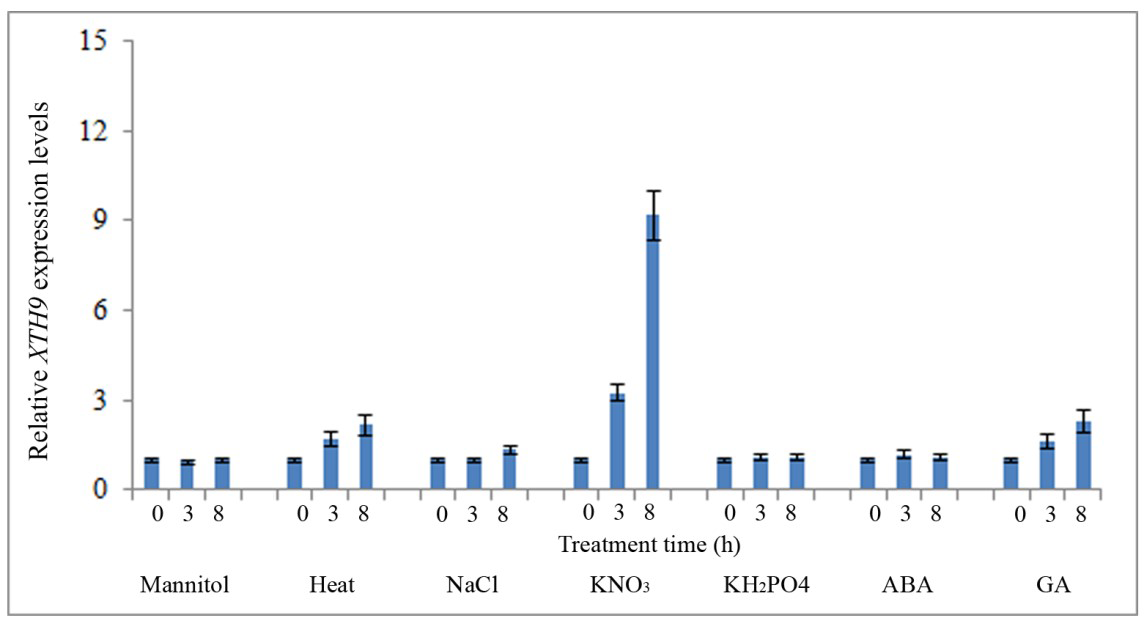

Supplement: S4 Fig — Ten-day-old wild-type plants grown on half-strength MS-agar plates were treated with 200 mM mannitol, heat (30°C), 100 mM NaCl, 500 μM KNO3, 1 mM KH2PO4 and plant growth hormones (1 μM ABA and 20 μM GA) for 3 and 8 hours. For drought treatment, the plants were transferred to dry 3M paper for 3 and 8 hours. RT-qPCR was used to check XTH9 expression levels at various time points. The error bars show the SDs (n = 3). (TIF) [file pgen.1008465.s004.tif]

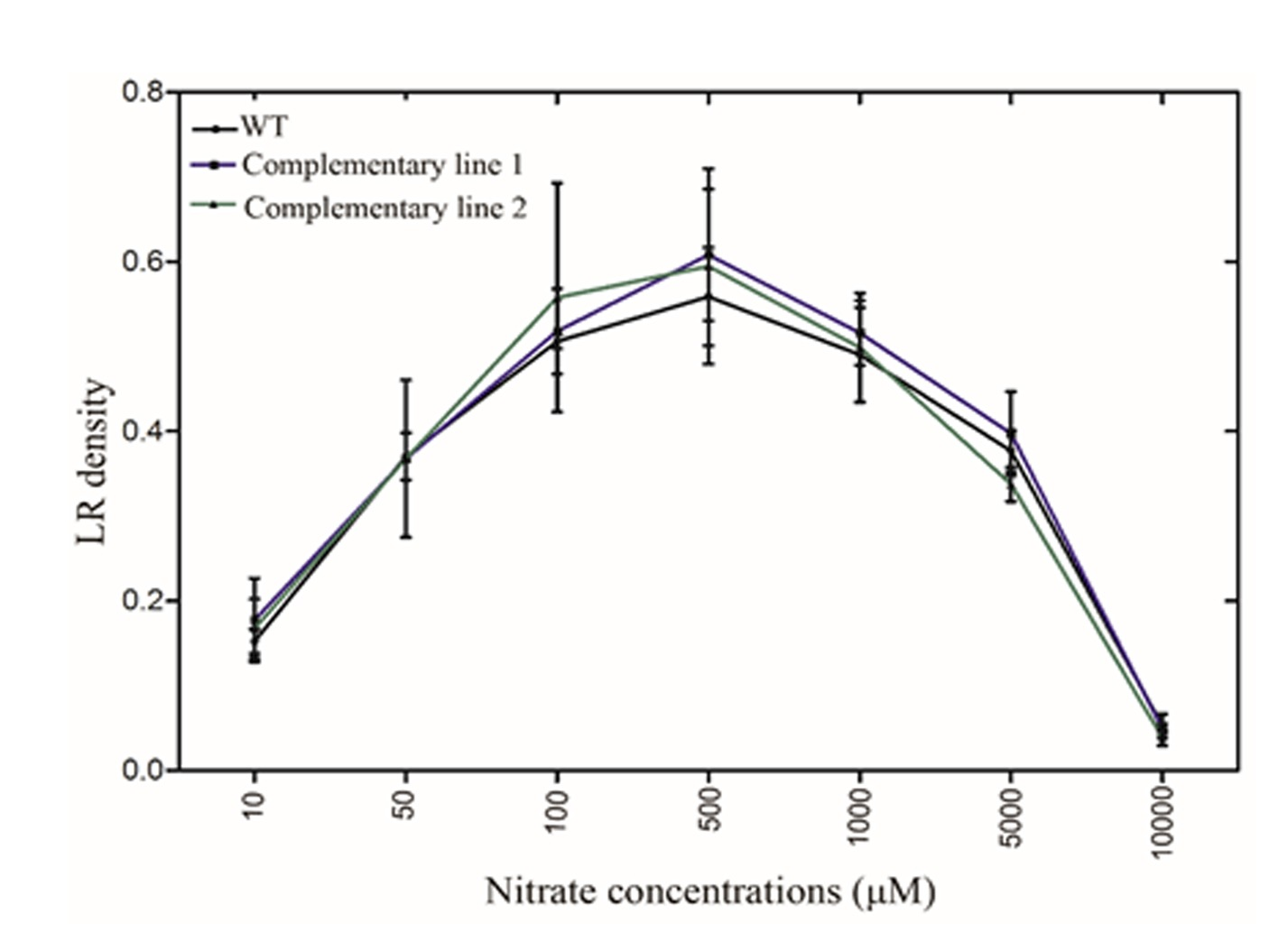

Supplement: S5 Fig — LR density (number of LRs per 1 cm of primary root length) in WT and complimentary lines grown in media supplemented with various concentrations of nitrate. The error bars show the SDs (n = 3). (TIF) [file pgen.1008465.s005.tif]

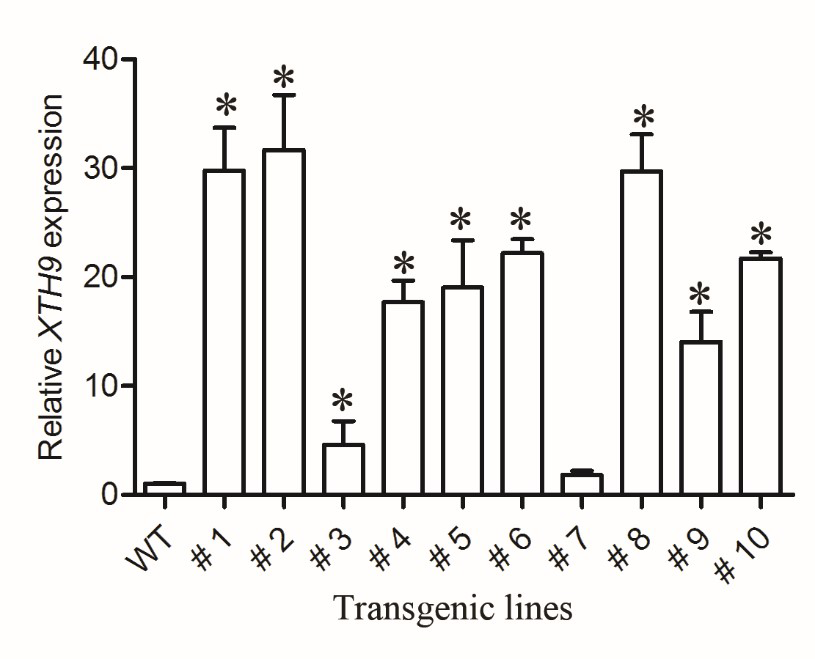

Supplement: S6 Fig — XTH9 expression level in the 2-week-old 35S::XTH9 transgenic plant leaves. *indicates significant differences (p<0.05). The error bars show the SDs (n = 3). (TIF) [file pgen.1008465.s006.tif]

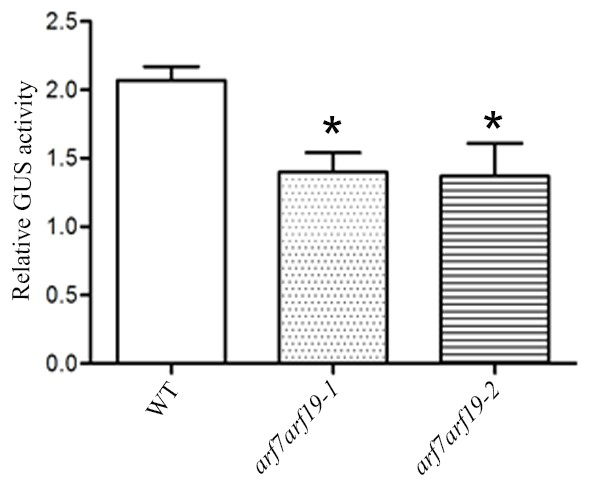

Supplement: S7 Fig — *indicates significant differences (p<0.05), and the error bars show the SD (n = 3). (TIF) [file pgen.1008465.s007.tif]

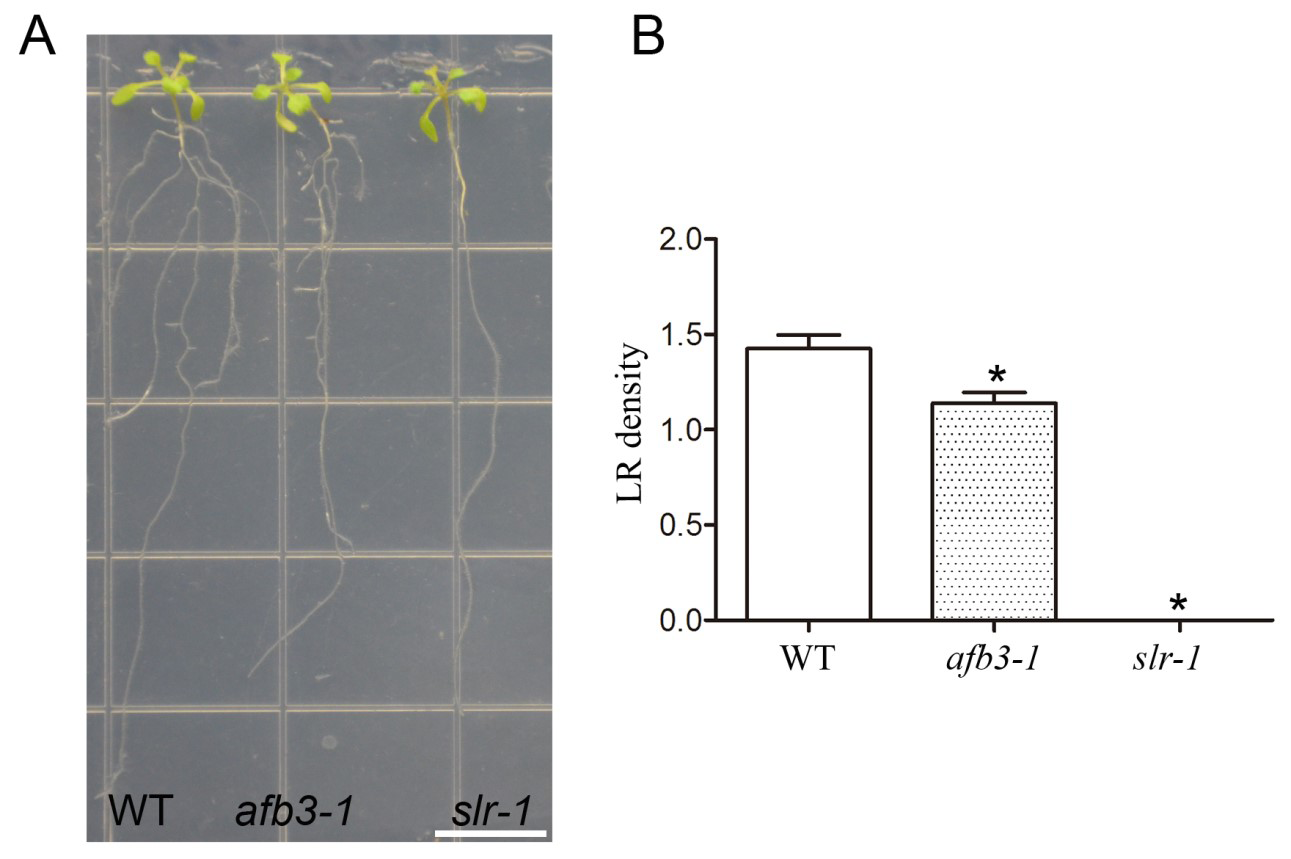

Supplement: S8 Fig — (A) Observations of wild-type, afb3-1 and slr-1 plant root development in response of 500 mM nitrate treatment for 2 day. The bar indicates 1 cm. (B) Analysis of LR density (LR per 1 centimeter of primary root). *indicates significant differences (p<0.05), and the error bars show the SD (n = 3). (TIF) [file pgen.1008465.s008.tif]

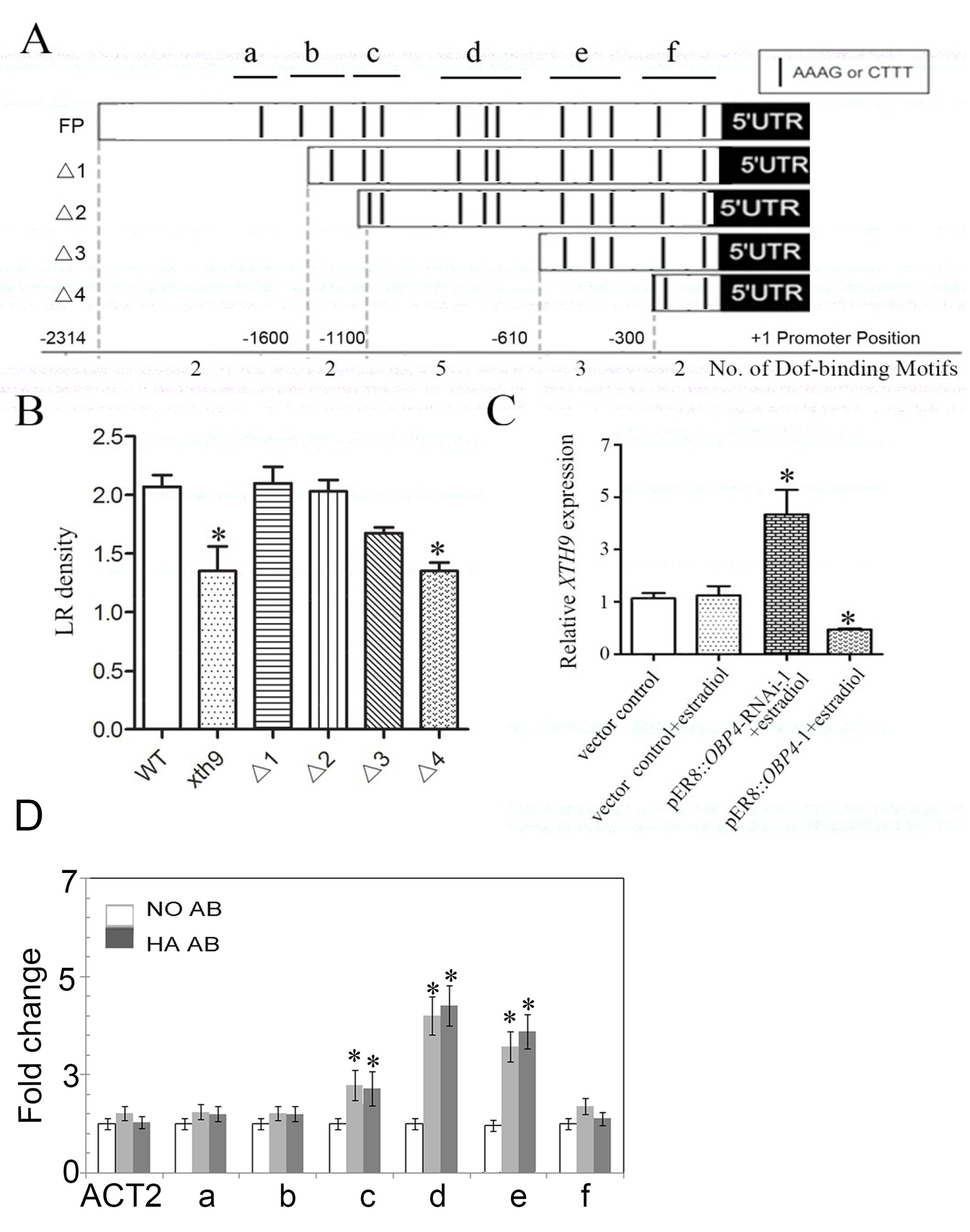

Supplement: S9 Fig — (A) The PLACEcare online tool was used to search for motifs. Many motifs including GATA-box-binding elements, the W-box elements and Dof TF-binding elements were found in the XTH9 promoter. Representation of the XTH9 full promoter from -2314 bp to the start codon (ATG). Promoter deletions (named Δ1, Δ2, Δ3 and Δ4) were generated and cloned upstream of XTH9::GUS. Dof-binding motifs are indicated as vertical solid lines. The sequence areas used for the ChIP experiment are marked in the gene promoters (from a to f, top panel). The number of Dof-binding motifs is indicated for each promoter deletion below the panel. (B) LR density measurements (number of LR per 1 cm of primary root length). The error bars represent the SDs. (n≥10). (C) Relative XTH9 activity in pER8 vector transgenic lines and inducible OBP4 expression and RNAi lines before and after 20 μM estradiol induction for 2 days. *indicates significant differences (p<0.05), and the error bars show the SD, n = 3. (D) 8-day-old pER8::OBP4::HA transgenic plants grown on MS-agar plates were used for ChIP assays. The enrichment shown was calculated as the DNA level of each fragment in the β-estradiol-treated sample divided by that in the DMSO-treated sample. Anti-HA antibody was used to precipitate OBP4-HA. Three measurements were averaged for individual assays. Bars indicate the SD. The values in Col-0 plants were set to 1 after normalization to ACT2 for qPCR analysis. Asterisks indicate significant differences, p< 0.05. (TIF) [file pgen.1008465.s009.tif]

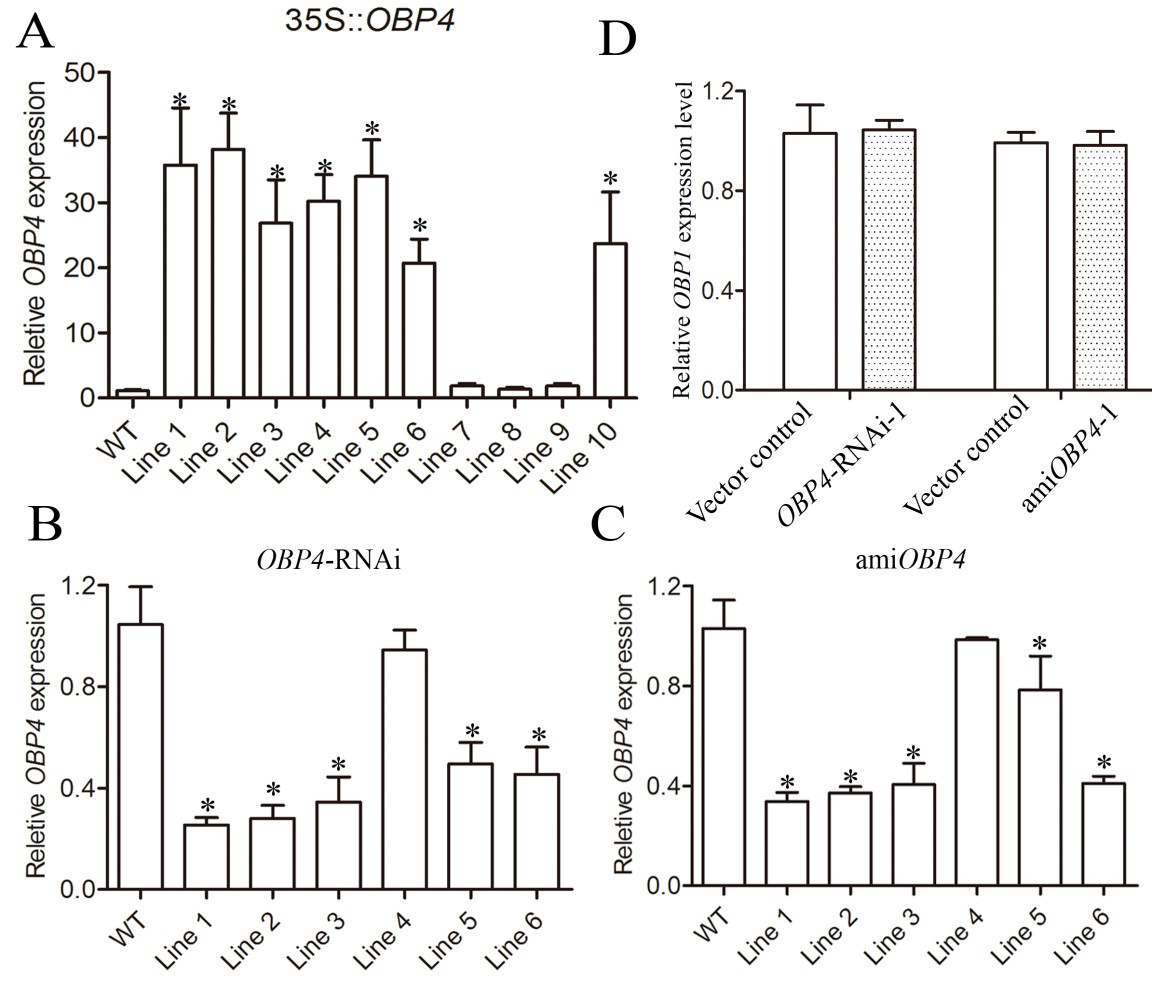

Supplement: S10 Fig — (A-C) Relative OBP4 expression levels in the 2-week-old 35S::XTH9, RNAi-OBP4 and amiOBP4 transgenic plants leaves. (D) OBP1 expression levels in the vector control and RNAi-OBP4-1 and amiOBP4-1 transgenic plants. *indicates significant differences (p<0.05). The error bars show the SDs (n = 3). (TIF) [file pgen.1008465.s010.tif]

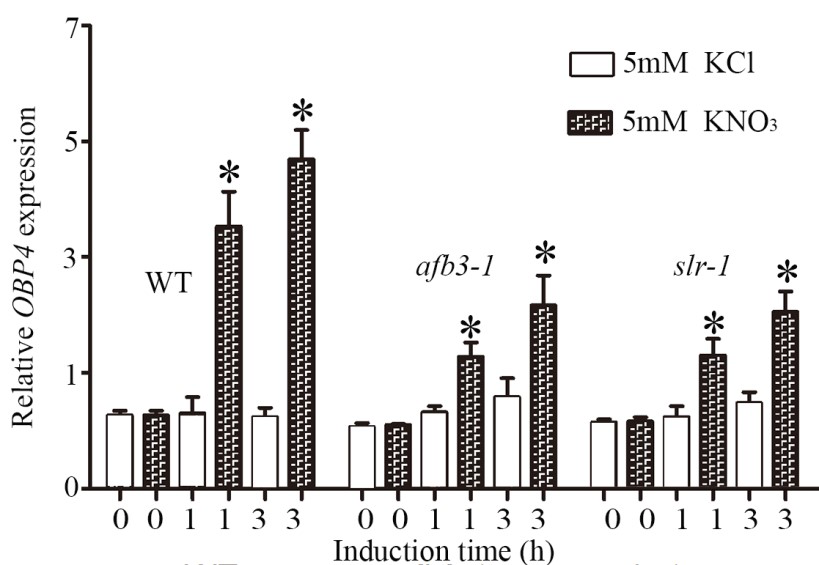

Supplement: S11 Fig — WT (Col-0), afb3-1 and slr-1 mutant plants were grown in media supplemented with ammonium succinate for one week and subsequently treated with 5 mM KNO3 or 5 mM KCl for 1–3 hours. The OBP4 gene expression level in plant roots was measured via RT-qPCR. The KCl treatment results are shown with white bars, and the KNO3 treatment results are shown with black bars. *indicates significant differences (p<0.05), and the error bars show the SDs (n = 12–18). (TIF) [file pgen.1008465.s011.tif]

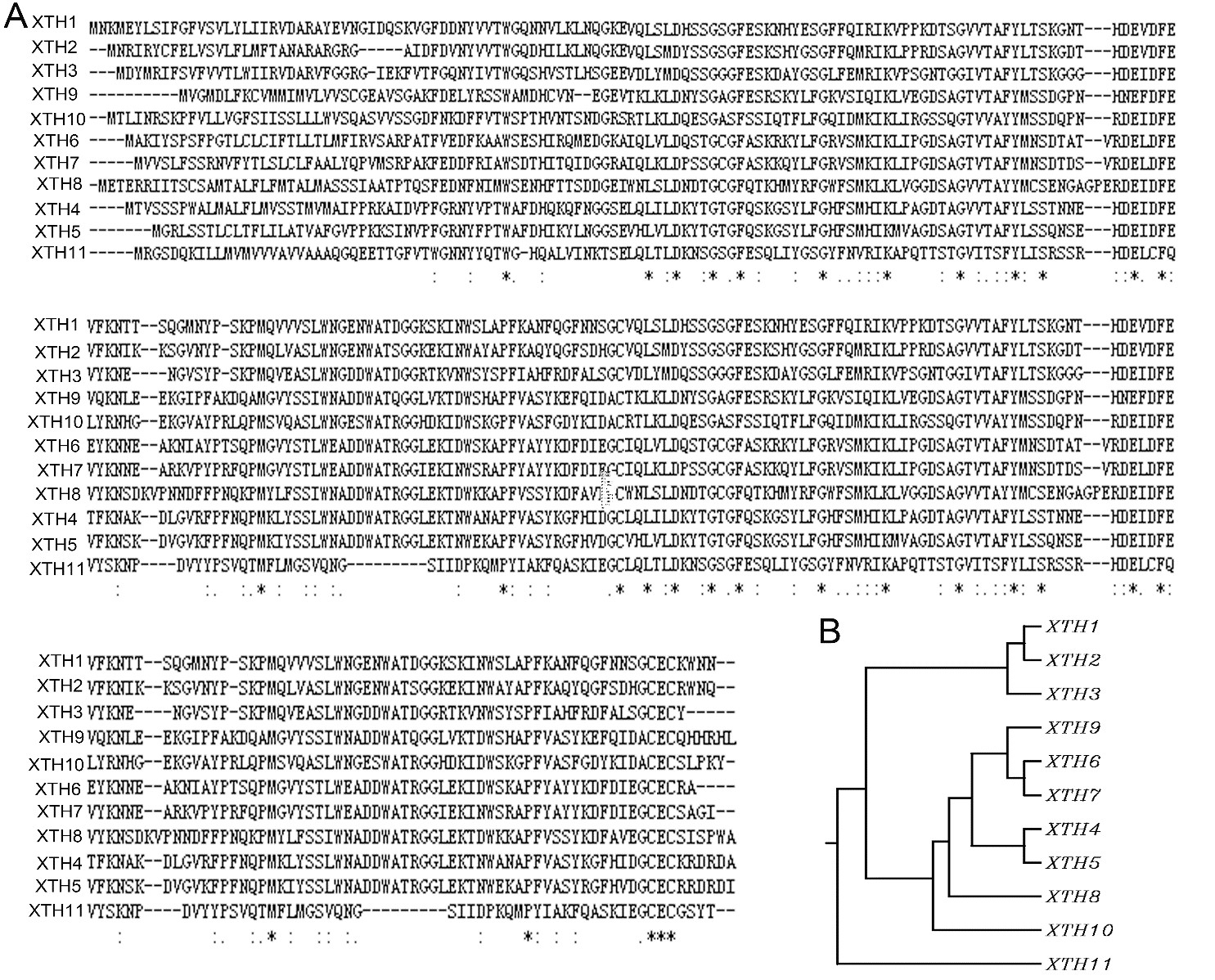

Supplement: S12 Fig — (A) Alignment and (B) Phylogram of Arabidopsis class 1 XTH family proteins. Multiple sequence alignment of the predicted amino acid sequence and phylogenetic analysis were performed via DNAMAN 6.0 and MEGA 4.1 software. (TIF) [file pgen.1008465.s012.tif]
