# Supplementary material for: Nitrate-responsive OBP4-XTH9 regulatory module controls lateral root development in Arabidopsis thaliana
Source: PLoS Genet. 2019 Oct 18;15(10):e1008465. doi: 10.1371/journal.pgen.1008465 (PMC6821136; doi:10.1371/journal.pgen.1008465)
Supplement: S1 Table — (DOCX) [file pgen.1008465.s013.docx]

**Table S1.** Primers used for plasmid construction and mutant isolation.

| **Transgenes** | **Primers (Sequence 5’-3’)** |
| --- | --- |
| Full-length *XTH9* promoter | 5'-AGTTTGAAGCTTTGTGATTAT-3′ |
|  | 5'-TTTTAACTTATCTCTCTAAATAAAT-3′ |
| Δ1*XTH9* promoter | 5'-TGGTCTGAAGACTGGAGGACGAA-3′ |
| Δ2*XTH9* promoter | 5'-AAAAATAAGAGCCGTTGGCTCAAAT-3′ |
| Δ3*XTH9* promoter | 5'-TTGAATTCCCCCAAAAAGTGA-3′ |
| Δ4*XTH9* promoter | 5'-GCTACATTTTTCTTTTATCTCTC-3′ |
| pHB::*XTH9* | 5'-G**AAGCTT** ATGGTCGGTA TGGATTTGTT-3′ |
|  | 5'-GG**TCTAGA** CAAATGACGATGATGTTGGC-3′ |
| pHB::*OBP4* | 5'-C**AAGCTT**ATGCAAGATATTCATGATTTC-3′^a^ |
|  | 5'-GG**TCTAGA**TCAAGGAAGGTAGAGACCAC-′^a^ |
| pER8::*OBP4*::OE | 5'-CC**CTCGAG** ATGGTCGGTA TGGATTTGTT-3′^a^ |
|  | 5'-GG**ACTAGT** CAAATGACGATGATGTTGGC-3′^a^ |
| SALK_118463 | 5'-ATTCCAAAAGAAGCTTCTCGC-3′ |
|  | 5'-CTGATGAGCTCTCATCCTTCG-3′ |
| SALK_116433 | 5'-ATTCCAAAAGAAGCTTCTCGC-3′ |
|  | 5'-TGAATTGGGAGGTTACAGTGG-3′ |
| SALK_101024 | 5'-GTTGGCTGATTTAAGCGTCAG-3′ |
|  | 5'-TGGTAGACGAAACACCGATTC-3′ |
| CS69190 | 5'-ATTCCAAAAGAAGCTTCTCGC-3′ |
|  | 5'-CTGATGAGCTCTCATCCTTCG-3′ |
| [SALK_061055](https://www.arabidopsis.org/servlets/TairObject?type=germplasm&id=3510706103) | 5'-TAGCCTTAAAGGGAGCCAAAG-3′ |
|  | 5'-AAACTTGCGTGTGGCTTAATG-3′ |
| SALK_028605 | 5'- CATGACGTGTCACTCAAATGG-3′ |
|  | 5'- AAATTGCCTCCAAGAGATTCC-3′ |
| [SALK_137968](https://www.arabidopsis.org/servlets/TairObject?type=germplasm&id=4855527) | 5'-TAAGGGATTTCGGTTAGGCAG-3′ |
|  | 5'-GGTTCCAAAGAAGCCCATAAG-3′ |
| [SALK_075813](https://www.arabidopsis.org/servlets/TairObject?type=germplasm&id=4678401) | 5'-AAAATGGCAGCACAAAATGAC-3′ |
|  | 5'-GCTGCTCTACGTAGGATGGTG-3′ |
| [SALK_085203](https://www.arabidopsis.org/servlets/TairObject?type=germplasm&id=4687791) | 5'-TGTATTTGACGCTCCTCCTTG-3′ |
|  | 5'-CATAAGAGGTGTTGCAGAGGC-3′ |
| [SALK_074666](https://www.arabidopsis.org/servlets/TairObject?type=germplasm&id=4677254) | 5'-CTCGAGTCGACCTTTGCTATG-3′ |
|  | 5'-TTTTGGAAGCAAATCCACAT-3′ |
| [SALK_201184](https://www.arabidopsis.org/servlets/TairObject?type=polyallele&id=502819023) | 5'-CTCTTATTACATGGCCCATGG-3′ |
|  | 5'-TACATACATTTGCCTGCATGC-3′ |
| [SALK_006889](https://www.arabidopsis.org/servlets/TairObject?type=germplasm&id=4517037) | 5'-TTTCGACCGACACAAAAATTC-3′ |
|  | 5'-TCGCTAATCCTAGGCCTATCC-3′ |
| SALK_001235 | 5'-TGGAAGCTTTTCTTTGGATTG-3′ |
|  | 5'-AGATTCATTCACATCAACCCG-3′ |
| SALK_045453 | 5'-AGATTTTGTGGCAACCACAAG-3′ |
|  | 5'-GATTCAAGCAGGAGACATTGC-3′ |
| *OBP4*::RNAi | 5'-CG **GGATCC CTAGA**TCCGAATATGGGATTTGAA-3′ |
|  | 5'-C **GAGCTC CTGCAG** ATTTGCACTCTTGGGATATT-3′ |
| Double 35S::*OBP4*::GFP | 5'-G**GAATTC** ATGCAAGATATTCATGATTTCTC-3′ |
|  | 5'-G**GTCGAC** AGGAAGGTAGAGACCACTCTGA-3′ |

Sequences shown bold are restriction enzyme digestion sites.
