# Supplementary material for: Nitrate-responsive OBP4-XTH9 regulatory module controls lateral root development in Arabidopsis thaliana
Source: PLoS Genet. 2019 Oct 18;15(10):e1008465. doi: 10.1371/journal.pgen.1008465 (PMC6821136; doi:10.1371/journal.pgen.1008465)
Supplement: S2 Table — (DOCX) [file pgen.1008465.s014.docx]

**Table S2. Gene-specific primers used in the qPCR experiments**

| Genes | Primers (Sequence 5’-3’) |
| --- | --- |
| *XTH9-1* | 5'-TGGTAGACGAAACACCGATTC-3’ |
|  | 5'-TCCTTGTGTAGCCCAATCATC-3’ |
| *XTH9-2* | 5'-ATTACTCTGGAGCTGGGTTTG-3’ |
|  | 5'-CGGACCATCTGAAGACATGTAG-3’ |
| *OBP4* | 5'-GAAGTTGCTTCGTCGGTTATTC-3’ |
|  | 5'-CGATGAGCCTTGTCCAAGTA-3’ |
| *NAC4* | 5'-CCAAGCTGAAACTAGAGGAACA-3’ |
|  | 5'-GAGGTTGGTAGAGTGGAATCAT-3’ |
| *ARF7* | 5'-TTTTCTGTACCCCGACGAGC-3’ |
|  | 5'- AGAGCCTTTTCGTGCTGACA-3’ |
| *ARF19* | 5'-GATTTCTCGATGCAACCGCC-3’ |
|  | 5'-ACGCTCCAACCTGTGGTAAG-3’ |
| *XTH17* | 5’-CGCTCGACAAATCCTCTGGA-3’ |
|  | 5’-CTCATCCCACGTAGTTCCCG-3’ |
| *ACTIN2* | 5’-TTCCCGTTCTGCGGTAGTGG-3’ |
|  | 5’-CCGGTATTGTGCTCGATTCTG-3’ |
| a | 5’-CTAGCTTTGTTTTTCTTTCTGTA-3’ |
|  | 5’- AAGAGAGACAAGGATACTCTC-3’ |
| b | 5’-CTATCTGCGAGATGAGAATATG-3’ |
|  | 5’- AAAAGAGCTAATTGGGGAAAT-3’ |
| c | 5’-GTTTTGTCTTTAAAAGCCTTA-3’ |
|  | 5’-TCAACCATCATGATCTTTGTT-3’ |
| d | 5’-TGATGGAGTATATAGTACCAAT-3’ |
|  | 5’-ACAAGTCCCACTCGTTGAAA-3’ |
| e | 5’-AACAATTTCGGATTAAGTAAA-3’ |
|  | 5’-GTGGCAATAAAATATCTTCTG-3’ |
| f | 5’-TCTTTCTACATTATTACTTCCA-3’ |
|  | 5’-GAGGTAAGGCTTTATAAAGG-3’ |
