# Supplementary material for: Nitrate-responsive OBP4-XTH9 regulatory module controls lateral root development in Arabidopsis thaliana
Source: PLoS Genet. 2019 Oct 18;15(10):e1008465. doi: 10.1371/journal.pgen.1008465 (PMC6821136; doi:10.1371/journal.pgen.1008465)
Supplement: S3 Table — (DOCX) [file pgen.1008465.s015.docx]

**Table S3.** **T-DNA insertion lines of class 1 *XTH* genes.**

| **Class 1 *XTH*s** | **Gene names** | **T-DNA mutants** |
| --- | --- | --- |
| *XTH1* | AT4G13080 | [SALK_061055](https://www.arabidopsis.org/servlets/TairObject?type=germplasm&id=3510706103) |
| *XTH2* | AT4G13090 | SALK_028605 |
| *XTH3* | AT3G25050 | [SALK_137968](https://www.arabidopsis.org/servlets/TairObject?type=germplasm&id=4855527) |
| *XTH4* | AT2G06850 | [SALK_075813](https://www.arabidopsis.org/servlets/TairObject?type=germplasm&id=4678401) |
| *XTH5* | AT5G13870 | [SALK_085203](https://www.arabidopsis.org/servlets/TairObject?type=germplasm&id=4687791) |
| *XTH6* | AT5G65730 | [SALK_074666](https://www.arabidopsis.org/servlets/TairObject?type=germplasm&id=4677254) |
| *XTH7* | AT4G37800 | [SALK_201184](https://www.arabidopsis.org/servlets/TairObject?type=polyallele&id=502819023) |
| *XTH8* | AT1G11545 | [SALK_006889](https://www.arabidopsis.org/servlets/TairObject?type=germplasm&id=4517037) |
| *XTH9* | AT4G03210 | [SALK_101024](https://www.arabidopsis.org/servlets/TairObject?type=germplasm&id=4818583), SALK_023274, SALK_002571 |
| *XTH10* | AT2G14620 | [SALK_001235](https://www.arabidopsis.org/servlets/TairObject?type=germplasm&id=4511383) |
| *XTH11* | AT3G48580 | [SALK_045453](https://www.arabidopsis.org/servlets/TairObject?type=germplasm&id=4648041) |
